# Development, Characterization, and Biological Evaluation of a Self-Healing Hydrogel Patch Loaded with Ciprofloxacin for Wound Dressings

**DOI:** 10.3390/polym17192686

**Published:** 2025-10-04

**Authors:** Wasan Al-Farhan, Osama H. Abusara, Mohammad Abu-Sini, Suhair Hikmat, Ola Tarawneh, Sameer Al-Kouz, Rania Hamed

**Affiliations:** Department of Pharmacy, Faculty of Pharmacy, Al-Zaytoonah University of Jordan, Amman 11733, Jordan; wasanmortada@gmail.com (W.A.-F.); o.abusara@zuj.edu.jo (O.H.A.); mohammad.abusini@zuj.edu.jo (M.A.-S.); suhair.jasim@zuj.edu.jo (S.H.); ola.tarawneh@zuj.edu.jo (O.T.); s.alkouz@zuj.edu.jo (S.A.-K.)

**Keywords:** ciprofloxacin, hydrogels, polymers, self-healing, wound dressing, wound healing

## Abstract

Hydrogels are crosslinked polymer chains that form a three-dimensional network, widely used for wound dressing due to their ability to absorb significant amounts of fluid. This study aimed to develop a hydrogel patch for wound dressing with self-healing properties, particularly for joints and stretchable body parts, providing a physical barrier while maintaining an optimal environment for wound healing. Polyvinyl alcohol (PVA) and sodium carboxymethyl cellulose (Na CMC) were crosslinked with borax, which reacts with the active hydroxyl groups in both polymers to form a hydrogel. The patches were loaded with ciprofloxacin HCl (CIP), a broad-spectrum antibiotic used to prevent and treat various types of wound infections. Hydrogels were subjected to rheological, morphological, antimicrobial, self-healing, ex vivo release, swelling, cytotoxicity, wound healing, and stability studies. The hydrogels exhibited shear-thinning, thixotropic, and viscoelastic properties. Microscopic images of the CIP hydrogel patch showed a porous, crosslinked matrix. The antimicrobial activity of the patch revealed antibacterial effectiveness against five types of Gram-positive and Gram-negative bacteria, demonstrating a minimum inhibitory concentration of 0.05 μg/mL against *E. coli*. The swelling percentage was found to be 337.4 ± 12.7%. The cumulative CIP release percentage reached 103.7 ± 3.7% after 3 h, followed by zero-order release kinetics. The stability studies revealed that the crossover point shifted toward higher frequencies after 3 months of storage at room temperature, suggesting a relaxation in the hydrogel bonds. The cytotoxicity study revealed that the CIP hydrogel patch is non-cytotoxic. Additionally, the in vivo study demonstrated that the CIP hydrogel patch possesses wound-healing ability. Therefore, the CIP PVA/Na CMC/Borax patch could be used in wound dressing.

## 1. Introduction

Skin is the largest protective organ system in the body. It is part of the immune system, serving as a chemical, physical, and bacterial barrier. It preserves water and prevents electrolyte loss, preventing dehydration. Additionally, it plays a crucial role in thermoregulation [1]. Skin wounds are injuries that disrupt the skin’s protective function, exposing the body to bacterial infections. The skin has a high capacity for self-regeneration [2]. However, some skin defects cannot heal spontaneously and require coverage and supervision.

Traditional wound dressings, such as gauze, are associated with significant problems. For instance, when wound dressing dries, it adheres to the wound. This adherence makes removal painful and delays wound closure due to the secondary damage it causes [3]. Nowadays, modern wound dressings are surpassing traditional dressings in many aspects. Firstly, they maintain moisture around the wound, promoting the growth of new tissues and facilitating the healing process by retaining the wound’s exposure to pro-healing cytokines. Secondly, they prevent infections by physically covering the wound and incorporating materials that inhibit bacterial growth. Moreover, modern wound dressings are non-adherent, allow for gas exchange, protect wounds from trauma, possess favorable mechanical properties, and are biodegradable, biocompatible, and elastic [4].

Among modern wound dressings, the most commonly used types are hydrogels, hydrocolloids, alginates, foams, and films [3]. These dressings can be medicated with antibiotic-incorporated dressings due to the risk of wound infections, or they can be non-medicated. For instance, the worldwide incidence of diabetic foot infection has been recorded to be 25–58%, with a significant amputation risk when certain risk factors are present [5]. Delivering the antibacterial agent directly to the wound site is a strategy aimed at reducing bacterial resistance [6]. Moreover, local therapies minimize potential systemic toxicity and preserve the gastrointestinal microbiota [7].

Hydrogels are three-dimensional (3D) networks formed by crosslinking hydrophilic polymeric materials. What is exceptional about hydrogels is their high water content (70–90%), which maintains a moist environment suitable for wound healing and provides a cooling effect that helps relieve pain [8]. Research has shown that a moist environment is ideal for wound healing, particularly for chronic wounds. Additionally, the healing rate is three to five times higher in a wet environment compared to a dry one [9]. There are several forms of hydrogels being formed for different purposes as well [10,11].

Hydrogels are divided into two types based on the type of bonding within the network: physical and chemical. A chemical hydrogel is formed when a crosslinker is added, resulting in the formation of covalent bonds between the polymer chains. In contrast, a physical hydrogel is formed when the polymer chains engage in hydrogen bonding, ionic interactions, or other physical forces [12]. Physically crosslinked hydrogels are easier to synthesize and do not require a chemical crosslinker. However, unlike chemically crosslinked hydrogels, they are more easily disturbed [13].

A type of hydrogel called “Self-healing hydrogels” can spontaneously repair its bonds after mechanical damage [14]. The dynamic chemical crosslinking reaction, such as disulfide bonds, imine bonds, and diol-borate ester bonds, is mainly responsible for the self-healing ability [14]. Moreover, physical interactions, such as hydrogen bonds, can aid in self-healing properties but require a longer duration to reform than chemically crosslinked hydrogels. However, physical interactions significantly enhance the strength of the healed gel. Combining both chemical and physical interactions improves the mechanical properties of the hydrogel [15].

Self-healing hydrogels have attracted significant interest due to their potential applications. They can be injected into specific body sites to fill irregular cavities or utilized as a drug delivery system for local treatments of tumors and wounds [16]. Additionally, they could be a promising option for wound dressings. With their durable mechanical properties, this smart material has surpassed other classes of modern wound dressings. For instance, the self-healing hydrogels can alter their shape and reorganize their bonds, rendering the hydrogel patches suitable for various body parts, including the face, elbows, knees, neck, and finger joints. This feature enables patients to feel more comfortable while the patch is applied to the wound.

To the best of our knowledge, this is the first study to report the preparation of a ciprofloxacin-loaded wound dressing patch using polyvinyl alcohol (PVA) and sodium carboxymethyl cellulose (Na CMC). PVA and Na CMC were physically crosslinked by hydrogen bonds and chemically crosslinked by reversible diol-borate ester bonds, using borax as an environmentally friendly and economical crosslinker, to prepare affordable hydrogels with good drug loading capacity and enhanced mechanical properties.

## 2. Materials and Methods

### 2.1. Materials

Ciprofloxacin HCl (CIP, Zhejiang Huayi Pharma Co., Yiwu, China) was kindly provided by Hikma Pharmaceuticals (Amman, Jordan). Poly (vinyl alcohol) (PVA, molecular weight (MW) = 13,000–23,000 g/mol, 87–89% hydrolyzed) was purchased from Sigma-Aldrich (St. Louis, MO, USA). Carboxymethyl cellulose sodium salt (Na CMC) (average MW = 90,000 g/mol, degree of substitution = 0.7) was purchased from Thermo Scientific (Waltham, MA, USA). Di-sodium tetraborate anhydrous LR was purchased from GHTECH (Shantou, China). Nutrient agar was purchased from NA, Biolab (Budapest, Hungary), while Mueller–Hinton agar medium and Nutrient Broth were sourced from HIMEDIA (Mumbai, India). Phosphate-buffered saline (PBS) tablets were purchased from Loba Chemie Pvt Ltd. (Mumbai, India). Dulbecco’s Modified Eagle Medium (DMEM) and Dulbecco’s Phosphate-Buffered Saline (PBS) were obtained from Euroclone (Pero, Italy). Heat-inactivated Fetal Bovine Serum (FBS) was acquired from Capricorn Scientific (Ebsdorfergrund, Germany). Primary Dermal Fibroblast: Normal, Human, Adult (HDFa) (ATCC PCS-201-012) was acquired from ATCC^®^ (Manassas, VA, USA). Resazurin sodium salt was acquired from Sigma-Aldrich (St. Louis, MO, USA). Diethyl ether was purchased from Tedia (Fairfield, OH, USA), and ultrapure water was generated using the EMD Millipore Direct-Q 5UV system (Darmstadt, Germany). All materials were used as supplied.

### 2.2. Methodology

#### 2.2.1. Solubility Study of Ciprofloxacin HCl in Polymer Solutions

A solubility study of CIP was conducted in Na CMC and PVA solutions to determine the maximum concentration of CIP that could be dissolved in the patch without precipitation. Various CIP concentrations of 0.2, 0.5, 1, 1.5, 3, 4, 5, and 6 mg/mL were prepared in (2.5, 3.5, and 5% Na CMC) or (10, 15, and 20% PVA). Ultrapure water was used as the solvent. The solutions were inspected the following day for any signs of precipitation.

#### 2.2.2. Preparation of Ciprofloxacin Hydrogels

Three solutions were used to prepare CIP hydrogel patches. The first solution was a 15% PVA solution containing 0.1% CIP (CIP-PVA). This solution was prepared by dissolving 100 mg of CIP in 92 mL of ultrapure water, stirring until it was fully dissolved. Afterward, 15 g of PVA was gradually added to the continuously stirred solution and left for about 2 h at 90 °C until fully dissolved. The volume was adjusted to 100 mL, resulting in a CIP solution of 1 mg/mL. The second solution was a 2.5% Na CMC solution, prepared by dissolving 2.5 g of Na CMC in 100 mL of ultrapure water at 60 °C, and stirring for approximately 2 h to ensure complete dissolution. The third solution was the borax solution, where three concentrations (1.5, 3, and 5%) were prepared by dissolving 1.5, 3, and 5 g of borax in 100 mL of ultrapure water at 100 °C, respectively, to determine the optimal concentration of borax for preparing CIP hydrogel patches.

The CIP hydrogel patch was prepared as described in [17]. To prepare 20 g of CIP hydrogel patch, 5 mL of 2.5% Na CMC solution and 12 mL of CIP-PVA solution were placed in a beaker and stirred at 70 ± 5 °C for 30 min. Subsequently, 4 mL of 1.5, 3, and 5% borax solutions were added dropwise to the continuously stirred mixture. After some time, crosslinks formed, resulting in a thick gel. The resulting gel was poured into a 9 cm diameter Petri dish and left to cool before being tightly sealed with Parafilm. Figure S1 illustrates the preparation method of CIP hydrogel patches.

#### 2.2.3. Attenuated Total Reflection-Fourier Transform Infrared Spectroscopy (ATR-FTIR) Analysis

FTIR spectra were obtained using ATR-FTIR (Vertex 70 spectrometer, Bruker, Ettlingen, Germany) for the pure drug powder (CIP), pure polymers (PVA and Na CMC), the crosslinker (borax), and the physical mixture of the hydrogel components at the same ratios of polymers, crosslinker, and drug in the hydrogel patch, as well as for the CIP hydrogel patch (12 mg CIP, 125 mg Na CMC, 1800 mg PVA, and 120 mg borax). The corresponding absorption spectra were recorded in the range of 4000–400 cm^−1^. The obtained spectral data were analyzed using the software Ira FTIR Data Explorer V 1.0. [18].

#### 2.2.4. Characterization of CIP Hydrogel Patches

##### UV–Vis Spectroscopic Analysis

Preparation of CIP calibration curve

A calibration curve for CIP was generated using a UV-vis spectrophotometer (UV–1800 spectrophotometer, Shimadzu, Kyoto, Japan) at a maximum wavelength (λ_max_) of 270 nm. A standard stock solution of 1 mg/mL was prepared with ultrapure water. Subsequently, a serial dilution was performed to obtain CIP concentrations ranging from 0.15 to 10 μg/mL to construct the CIP calibration curve. Three replicates were conducted.

Drug Recovery of CIP from the CIP hydrogel patches

The amount of CIP in the CIP hydrogel patches was determined using UV-vis spectrophotometry at a λ_max_ of 270 nm. A pre-weighed piece of approximately 200 mg of the CIP hydrogel patch was placed in 20 mL of ultrapure water and sonicated at 35 °C for 20 min. The sample was then appropriately diluted with ultrapure water, and the concentration of CIP in the CIP hydrogel patch was assessed by measuring the absorbance at 270 nm. The test was conducted in triplicate. The drug recovery percentage was calculated using Equation (1):

Drug Recovery% = (Amount of drug recovered/Theoretical drug amount) × 100%(1)

##### Rheological Studies

Rheological measurements were determined using a controlled-stress rheometer (CSR) (Anton Paar, MCR 302; Graz, Austria) with a parallel plate-plate geometry featuring a 25 mm diameter and a 1° cone angle. The gap between the parallel plate and the lower plate was 1 mm. All measurements were conducted at 32 °C to mimic skin conditions [19].

Viscosity

The viscosity studies of the CIP hydrogel patches were conducted at 32 °C to mimic skin conditions as described in [20]. A 1.5 cm^2^ sample of the CIP hydrogel patch was carefully placed on the lower plate to minimize shearing during sample loading. The viscosity of the hydrogels was measured at shear rates ranging from 0.1 to 100 s^−1^. The viscosity study was conducted on a fresh sample (prepared after 1 h) and after 24 and 48 h, with at least three replicates at each interval.

Strain-sweep studies

Strain-sweep studies were conducted on the CIP hydrogel patches to determine the linear viscoelastic region (LVR), as described in [20]. The frequency was kept constant in this test, while the oscillatory strain increased gradually. The storage modulus G′ and loss modulus G″ were determined over a strain range of 0.01–100%. A 1.5 cm^2^ piece of CIP hydrogel patch was placed on the lower plate, and the parallel plate was oscillated at a constant frequency of 6.28 rad/s. This study was conducted on fresh hydrogel (prepared after 1 h) that was allowed to crosslink for 24 and 48 h, with at least three replicates for each time point.

Frequency-sweep studies

The frequency sweep test was conducted on the CIP hydrogel patches at 32 °C to evaluate the viscoelastic properties (G′ and G′′) as described in [20]. The test was conducted over a frequency range of 0.1–100 rad/s, with a constant strain selected within the LVR based on the amplitude-sweep study. A 1.5 cm^2^ piece of the CIP hydrogel patch was placed on the lower plate, and the parallel plate was oscillated at different frequencies. This study examined fresh hydrogel (prepared after 1 h) that was allowed to crosslink for 24 and 48 h, with at least three replicates at each time point.

**Three-interval thixotropy test** (**3ITT**)

The self-healing ability of the CIP hydrogel patches was examined using the three-interval thixotropy test (3ITT) to assess the ability of the hydrogel bonds to recover after deformation. Initially, a 1.5 cm^2^ piece of the CIP hydrogel patch crosslinked for 24 h was subjected to a low shear strain (oscillating) of 0.1%, followed by a high shear strain of 100%, and then another low shear strain of 0.1%, as described in [21]. The test was conducted in triplicate at an angular frequency of 10 rad/s. The changes in complex viscosity and recovery time are reported.

##### Morphology of CIP Hydrogel Patch

Scanning electron microscopy (SEM) analysis was performed using a high-resolution field-emission scanning electron microscope (SEM, Apreo 2S LoVac, Thermo Fisher Scientific) under an accelerated voltage of 10 kV for secondary electron imaging to acquire surface topography images of the crosslinked hydrogel. CIP hydrogel samples were freeze-dried and coated with a 4–6 nm carbon layer using pulsed rod evaporation to render the sample conductive. Various magnifications were used to image the hydrogel.

##### Swelling Behavior of CIP Hydrogel Patches

The swelling behavior of CIP hydrogel patches was studied in PBS (pH 7.2) at room temperature as described in [22]. A pre-weighed piece of completely dried hydrogel was placed in 15 mL of PBS. At designated time intervals, the swollen hydrogels were removed from the buffer, and the excess solution was wiped off using filter paper. The swollen hydrogel was then weighed using an analytical balance. The swelling ratio was calculated using Equation (2) [22]:

Swelling index = (W_s_ − W_d_)/W_d_ × 100 (2)

where W_s_ is the weight of the swollen hydrogel and W_d_ is the weight of the completely dried hydrogel.

##### Self-Healing Analysis

Two CIP hydrogel patches were prepared to test their self-healing ability as described in [23]. The first patch was stained with food colorant, whereas the second patch remained uncolored. A piece of each patch was placed in contact with the other and allowed to heal without any applied stress.

##### Stability Studies of CIP Hydrogel Patch

After one and three months of storage at room temperature, the physical stability of the CIP hydrogel patches was assessed. This included appearance, transparency, and checking for the precipitation of CIP. Additionally, the rheological stability, including viscosity, the linear viscoelastic region (LVR), and viscoelastic properties (G′ and G′′), along with healing ability, stretchability, and drug recovery, were evaluated after one and three months of storage at room temperature to understand the structural and mechanical changes of the hydrogel.

#### 2.2.5. Antimicrobial Study

##### Well-Diffusion Method

The antimicrobial activity of the CIP hydrogel patches was assessed against Gram-positive and Gram-negative bacteria using the well-diffusion method, as described in [20]. Various concentrations of CIP (0.01, 0.02, and 0.05%), which are equivalent to 110, 250, and 570 μg/mL, were utilized to identify the optimal concentration that does not precipitate over time and is effective against bacteria. The microorganisms used in this study, obtained from ATCC, were initially subcultured onto nutrient agar and incubated at 37 °C to obtain isolated colonies. Bacterial inocula were prepared in 5 mL of nutrient broth (0.5 McFarland standards correspond to 1.5 × 10^8^ CFU/mL).

Five species of bacteria were used to assess the antimicrobial activity of the CIP hydrogel patches: three Gram-negative bacteria (*Escherichia coli* (*E. coli*) ATCC 8739, *Pseudomonas aeruginosa* (*P. aeruginosa*) ATCC 9027, and *Proteus mirabilis* (*P. mirabilis*) ATCC 12453), and two Gram-positive bacteria (*Bacillus spizizenii* (*B. spizizenii*) ATCC 6633 and *Staphylococcus aureus* (*S. aureus*) ATCC 6538). A Gram stain test was conducted to verify the purity of the bacterial inoculum for each bacterial species. Mueller–Hinton agar was used as the medium. A volume of 20 mL of the sterilized medium was poured into Petri dishes and allowed to solidify. Subsequently, 100 μL of microbial culture was evenly spread on the Mueller–Hinton agar plates using a sterile culture swab. Wells with a 6 mm diameter were aseptically made on the agar plates.

A CIP solution of 5 μg/mL was used as a positive control, while distilled water (DW) and blank hydrogel patches served as negative controls. Pieces of CIP hydrogel patches, each 6 mm in diameter and containing three different concentrations of CIP (0.01, 0.02, and 0.05%), along with a blank hydrogel patch, were carefully placed into the wells. A total of 50 μL of distilled water and CIP solution were used to fill the wells. The culture plates were incubated at 37 °C for 18 to 24 h, and the results were recorded the following day. The zones of inhibition created around the wells were measured using a centimeter ruler. The test was performed in triplicate.

##### Determination of Minimum Inhibitory Concentration (MIC)

A well-diffusion method was used to determine the minimum inhibitory concentration (MIC) of CIP in the CIP hydrogel patch. Fifteen serial dilutions of CIP loaded into the CIP hydrogel patches (ranging from 0.02 to 570 μg/mL) were prepared and tested using the well-diffusion method described in the section above on the same five bacterial species. Three replicates were conducted for each concentration of each bacterial species.

#### 2.2.6. In Vitro Cytotoxicity Study

To assess the safety of the CIP hydrogel, a cytotoxicity assay was conducted on the primary human dermal fibroblast (HDFa) cell line using the resazurin dye colorimetric method, as described in [24,25]. HDFa cells were grown in Dulbecco’s Modified Eagle Medium (DMEM) supplemented with 10% *v*/*v* Fetal Bovine Serum (FBS) and 1% *v*/*v* Penicillin (10,000 U/m)-Streptomycin (10 mg/mL), forming the complete medium (10% *v*/*v* FBS/DMEM). The cells were incubated at 37 °C in a humidified air atmosphere of 5% CO_2_. HDFa cells are adherent and were continuously washed with PBS and supplemented with fresh complete medium to maintain their growth in a T75 cell culture flask. When the cells reached 70–80% confluency, they were washed with PBS. Afterward, 2 mL of trypsin/EDTA was added, and the flask was incubated for 3–4 min to detach the cells. The detachment was confirmed using a microscope (ZEISS Primovert, Oberkochen, Germany), and the trypsin was deactivated with 10 mL of the complete medium. The cells were counted using a hemocytometer and Trypan blue (HyClone^TM^ from Cytiva, Marlborough, USA, USA). A mixture of 10 μL of cell suspension and 10 μL of Trypan blue was prepared. From this mixture, 10 μL was added to the hemocytometer. Under the light microscope (ZEISS Primovert Microscope, Oberkochen, Germany), the cells in the 4 corner squares of the hemocytometer were counted manually at 10× magnification, where living cells are colorless, and dead cells appear blue. The cell density was calculated using Equation (3):

Cell density (cell/mL) = Average of cell counted × DF × 10^4^ (3)

The CIP hydrogel patch and its respective blank hydrogel patch were prepared and tested for in vitro cytotoxicity. The preparation of the hydrogels for the in vitro cytotoxicity assay was performed as described in [23]. Briefly, 0.1 g of the hydrogels was sterilized for 30 min using UV irradiation. The hydrogels were then immersed in a sterile tube containing 5 mL of complete medium for 24 h at 37 °C for hydrogel extraction. Following this, the tubes were centrifuged for 5 min at 120× *g*, and the extracts (media) were collected and filtered through a 0.22 µm ExtraGENE^®^ Syringe Filter (ExtraGene Inc., Taiwan, China), resulting in stock extracts with a concentration of 20 mg/mL. A serial 1:1 dilution was prepared using the complete medium to obtain diluted extract concentrations of 10 to 0.078 mg/mL, which were used in the in vitro cytotoxicity assay. The cytotoxicity assay was conducted using the resazurin dye colorimetric method. Briefly, HDFa cells were seeded in 96-well plates at a seeding density of 10,000 cells/200 μL/well in triplicate. The cells were allowed to adhere for 24 h during incubation. The media was aspirated, and the extracts (200 µL), with the different concentrations prepared above, were added to each well in triplicate. The plates were then incubated for 24 h. Untreated control samples were also prepared, in which the complete medium (200 μL) was added. Samples for background fluorescence for the resazurin dye were also prepared using only complete medium (200 μL). After 24 h of incubation, resazurin dye (20 μL; 0.125 g/L in PBS) was added to each well, and the plates were incubated for 2 h. Fluorescence readings (excitation 540 nm/emission 620 nm) were recorded using a BioTeK SYNERGY HTX multi-mode plate reader (Agilent Technologies, Inc., Santa Clara, CA, USA). Assays were performed in triplicate in three independent trials. The readings were analyzed to calculate the percentage viability relative to controls using GraphPad Prism version 9.0.

#### 2.2.7. Bioadhesion Study of CIP Hydrogel Patch

The bioadhesion test was conducted at room temperature using a TA-XT plus texture analyzer (Stable Micro Systems, Godalming, UK) in tension mode, with Texture Exponent 32 software (Hamilton, MA, USA). Firstly, rat skin was obtained from the back of a healthy Wistar rat in accordance with the approved protocols for rat skin preparation established by the Ethics Committee for Scientific Research at Al-Zaytoonah University of Jordan (approval number IRB No. 20/11/2024-2025). The hair was shaved, and the skin was cut into pieces with scissors; any excess fat tissue was also removed. The skin was frozen at −80 °C until the day of the experiment.

On the day of the experiment, the test setup was prepared as described in [26] with minor modifications. The skin was thawed at room temperature, and excess water was gently blotted away with a tissue. The skin was allowed to dry for a short time before the test began. Then, the skin was moistened with phosphate-buffered saline (PBS) and attached to the lower end of a cylindrical probe (5 cm in length and 1.5 cm^2^ in surface area) using metallic rings. A 2 cm^2^ piece of CIP hydrogel patch was placed below the probe on the plate (Figure S2). The probe was lowered until the rat skin and the hydrogel made contact. After 30 s of contact time, the probe was withdrawn upward at a speed of 0.5 mm/s. The detachment forces of CIP hydrogel patches crosslinked with 1.5, 3, and 5% borax were evaluated to assess the impact of borax concentration on the adhesion of the hydrogels. The resulting force–time curve was analyzed to determine the bioadhesion strength of each hydrogel. Three replicates of each hydrogel were tested.

#### 2.2.8. Ex Vivo Permeation

The ex vivo permeation study of the CIP hydrogel patches was conducted using Franz diffusion cells (PermeGear, Inc., Hellertown, PA, USA) with an effective surface area of 1 cm^2^ and an 8 mL receiver chamber. The study was conducted at 32 °C to simulate skin conditions in [20]. Following the approved protocols for rat skin preparation by the Ethics Committee for Scientific Research at Al-Zaytoonah University of Jordan (approval number: IRB No. 20/11/2024-2025), a full-thickness excised rat skin was obtained from the back of a rat. The skin was fixed between the donor and the receptor compartments using a clamp. A pre-weighed piece of the CIP hydrogel patch was placed on the stratum corneum (*SC*) of the skin in the donor compartment. The receptor compartment was filled with phosphate-buffered saline (PBS) (pH 6.5) and continuously stirred to simulate in vivo conditions. Subsequently, samples of 1 mL were withdrawn from the receptor compartment at the following time points: 0.25, 0.5, 1, 2, 3, and 4 h. A volume of 1 mL of fresh buffer was added to the receptor compartment at each time point to replace the withdrawn sample and maintain sink conditions. The amount of CIP was determined using a UV-vis spectrophotometer (Specord 200 plus Analytik, Jena, Germany) at 275 nm. The cumulative amount of CIP permeated through skin per unit surface area (Q/A) (A = 1 cm^2^, where A is the orifice surface area of the diffusion cell) was plotted versus time (t), and the steady-state flux (J_ss_, µg/cm^2^/h) was calculated from the slope of the linear portion of the (Q/A) versus (t) plot. The apparent permeability (P, cm/h) was calculated according to Equation (4) [25]:

P = Jss/Cο(4)

where Co is the concentration of the drug in the donor solution.

#### 2.2.9. In Vivo Wound Healing Study—A Pilot Study

This study assessed the effect of a CIP hydrogel patch on the wound healing process. Six male Balb/c mice (22–26 g) were divided into three groups, each containing two mice. The wounds in group 1 (negative control) received no treatment, while the wounds in group 2 were treated with CIP hydrogel patch, and the wounds in group 3 were covered with a blank hydrogel patch (the same patch used for group 2, but without CIP). The mice were isolated in solo plastic cages with unrestricted access to food and water. The animal study was conducted in accordance with animal ethics policies and received approval from the Animal Welfare Committee at Al-Zaytoonah University of Jordan (IRB No. 20/11/2024-2025). Mice were anesthetized using a ketamine injection, and an equal portion of the dorsum of each mouse was shaved. Afterward, minor superficial cuts were made to the skin using a sterilized blade, as described in [27]. After anesthetizing the mice with diethyl ether, the treatment was applied once daily, 5 days a week. A 1 g hydrogel sample was applied to the wound site and covered with a secondary dressing (Tegaderm, Saint Paul, MN, USA), which was fixed in place using adhesive tape. The cages were cleaned daily.

### 2.3. Statistical Analysis

The statistical analysis was conducted using GraphPad Prism 9.0 software. A one-way ANOVA with a multiple comparison test was utilized to compare the datasets of the cytotoxicity assay and the viscosity of the CIP hydrogel patch in the stability study. Statistical significance was determined based on *p*-value, where *p* < 0.05 was considered significant.

## 3. Results and Discussion

### 3.1. Solubility of Ciprofloxacin HCl in the Polymer Solutions

One of the biggest challenges in dealing with CIP is its solubility [20]. Serial concentrations of CIP in polymer solutions were tested to determine its solubility in the polymers used in this study. The drug was dissolved in ultrapure water, and then, different amounts of PVA or Na CMC polymers were added to achieve concentrations ranging from 0.2 to 6 mg/mL. Concentrations of 15% PVA and 2.5% Na CMC were chosen for the preparation of CIP hydrogel patches in this study, as they provide favorable mechanical properties in the resulting hydrogel. CIP precipitated after varying periods, from minutes to a week, in most of the prepared solutions. It was found that CIP dissolved in the Na CMC solution tended to precipitate at a lower concentration compared with CIP dissolved in the PVA solution (0.5 mg/mL vs. 1 mg/mL). The precipitation behavior of CIP in Na CMC solution has been reported in the literature [28]. Abdelkader et al. [28] proposed an electrostatic interaction between CIP, the hydrochloride salt of the weak base, and the sodium salt of CMC, which causes CIP to precipitate. Therefore, the highest CIP concentration that remained dissolved in the Na CMC solution without precipitating was 0.5 mg/mL. However, a concentration of 1 mg/mL CIP in the PVA solution was the maximum that did not precipitate in either the solution or the final hydrogel patch. As a result, a concentration of 1 mg/mL of CIP in a 15% PVA solution was chosen for the preparation of a hydrogel patch containing 0.05% CIP, representing the highest stable concentration of CIP in the hydrogel.

### 3.2. Ciprofloxacin Hydrogel Patches

**Figure 1:**
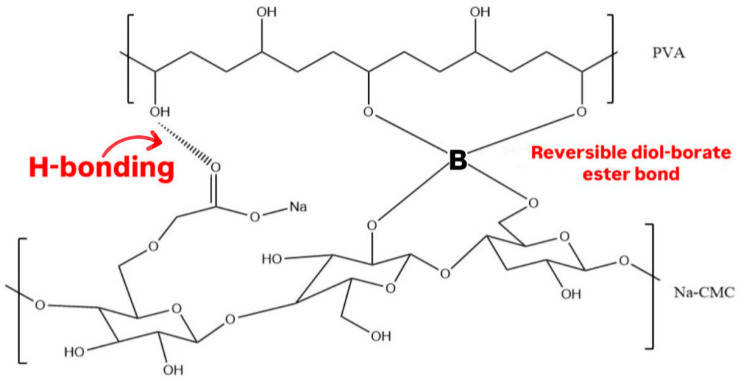
Hydrogen bonds between Na CMC and PVA and reversible diol-borate ester bonds between borax-Na CMC and borax-PVA.

The CIP hydrogel patches exhibited modified chemical and physical properties due to the double polymer network, as two polymers are crosslinked. PVA and Na CMC, which are miscible and compatible polymers, bind together through hydrogen bonds between the OH groups in PVA and the carboxymethyl groups and OH groups in CMC [29], as illustrated in Figure 1.

**Figure 2:**
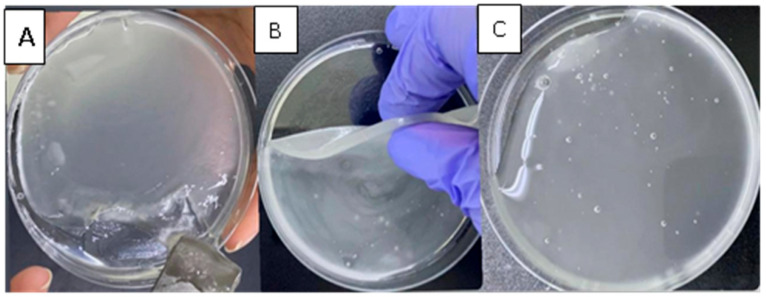
Hydrogels formed using various concentrations of borax solutions: (**A**) 1.5% borax solution formed a weak hydrogel, (**B**) 3% borax solution formed a hydrogel with a good structure, and (**C**) 5% borax solution formed a hydrogel that did not conform to the shape of the Petri dish after 24 h.

Following the addition of borax solutions (1.5, 3, or 5%), blank hydrogel patches formed due to the crosslinking between borate ions and OH groups in PVA and CMC chains [17], as illustrated in Figure 1. After 24 h, the three hydrogels were visually monitored for structural differences. The hydrogel crosslinked with a 1.5% borax solution exhibited a weak structure that could not be removed from the Petri dish as a single piece. In contrast, hydrogel crosslinked with a 5% borax solution displayed a rigid structure that did not relax and fill the Petri dish. The hydrogel crosslinked with a 3% borax solution demonstrated good mechanical properties during handling and relaxed to cover the Petri dish (took its shape) (Figure 2). Thus, a 3% borax solution was chosen to prepare the CIP hydrogel patch.

**Figure 3:**
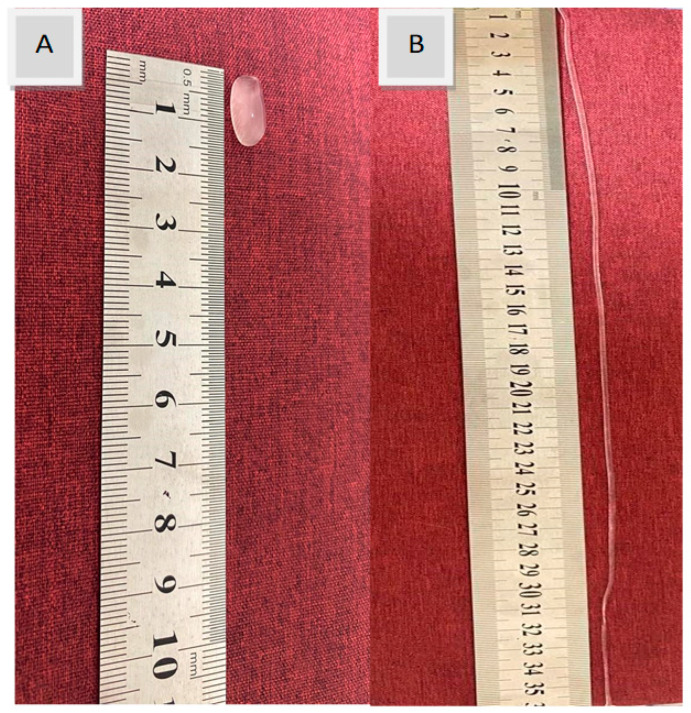
Stretchability of the CIP hydrogel patch: (**A**) before stretching and (**B**) after stretching. The hydrogel can stretch up to 35 times its original length without breaking.

The dynamic covalent network, formed by the boronic ester bonds, provides favorable characteristics for the hydrogel. A rearrangement of the bonds can occur in response to physical or chemical stimuli, such as mechanical load or temperature change, through the breakage and reformation of the bonds. Thus, this type of bond combines two crucial features: the strength of covalent bonds and the reversibility of non-covalent bonds [30]. These chemical and physical bonds confer unique mechanical properties to the hydrogel patch, including flexibility, self-healing ability, and stretchability, enabling the hydrogel patch to stretch up to 35 times its original length without breaking (Figure 3). The resulting CIP hydrogel patch is transparent, with no evidence of component separation or segregation.

### 3.3. Attenuated Total Reflection-Fourier Transform Infrared Spectroscopy (ATR-FTIR) Analysis

**Figure 4:**
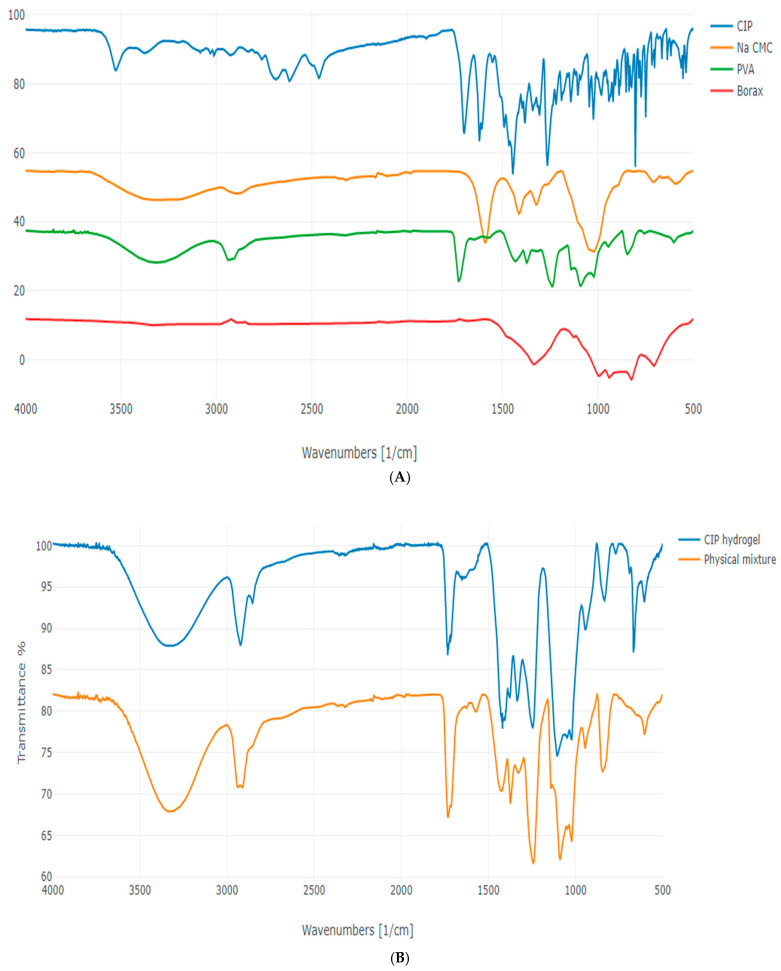
The FTIR spectra of: (**A**) CIP, Na CMC, PVA, and borax, and (**B**) CIP hydrogel patch and its corresponding physical mixture.

The FTIR spectra of CIP, Na CMC, PVA, and borax are presented in Figure 4A. The FTIR spectrum of CIP showed an OH stretching vibration peak at 3529 cm^−1^ and an OH bending vibration peak at 1265 cm^−1^, confirming the presence of a carboxylic acid. A band in the range of 2900–3000 cm^−1^ corresponds to the stretching of alkene and aromatic C–H bonds. The peak at 1702 cm^−1^ corresponds to the C = O stretching vibration, and the peak between 1650 and 1600 cm^−1^ refers to the NH bending of quinolones. A strong absorption peak between 1050 and 1000 cm^−1^ was assigned to the C–F group. The NH stretching vibration of the imino-moiety of the piperazine ring was observed at 3372 cm^−1^. These observations align with Sahoo et al. [31].

The Na CMC spectrum exhibited a broad peak at 3267 cm^−1^, corresponding to OH groups, and a peak centered at 2887 cm^−1^, corresponding to C–H stretching. Additionally, a sharp, well-defined peak was observed at 1591 cm^−1^ corresponding to the stretching of the –COO^−^ group of CMC. Bending vibration peaks of C–H bonds appeared between 1300 and 1450 cm^−1,^ and a band at 1024 cm^−1^ corresponds to CH–O–CH_2_ stretching. These FTIR observations align with Bucak and Sahin [17], who reported an OH group at 3341 cm^−1^, C–H stretching at 2886 cm^−1^, –COO^−^ group at 1589 cm^−1^, CH_2_ at 1421 cm^−1^, and OCHOCH_2_ at 1049 cm^−1^.

The FTIR spectrum of PVA displayed a characteristic broad band at 3312 cm^−1^, corresponding to the O–H stretching. The peak observed at 2937 cm^−1^ corresponds to the aliphatic C–H stretching vibrations. Additionally, the peaks observed at 1433 and 1730 cm^−1^ are attributed to the CH–OH and C = O groups, respectively. AÇIK et al. [32] showed a broad –OH band between 3000 and 3600 cm^−1^, corresponding to the intramolecular and intermolecular hydrogen bonding, and the bands at 2920 cm^−1^ and at 1425 cm^−1^ correspond to the –CH_2_ and CH–O–H, respectively.

The FTIR spectrum of borax (di-sodium tetraborate anhydrous) displayed an absorption band at 1324 cm^−1^, corresponding to the B–O asymmetric stretching in BO_3_ and aligning with Yong-Sing et al. [33]. The bands at 943 and 828 cm^−1^ correspond to the symmetric stretching of B–O in BO_3_ and BO_4_, respectively. Additionally, the bending of B–O was observed at 709 cm^−1^. These observations are consistent with Goel et al. [34], who reported symmetric stretching of B–O in BO_3_ and BO_4_ at 944 cm^−1^ and 826 cm^−1^, respectively, and out-of-plane bending of B–O in BO_3_ at 710 cm^−1^.

The FTIR spectra of the CIP hydrogel patch and its respective physical mixture are illustrated in Figure 4B. Both FTIR spectra exhibit several typical peaks, including the broad peak observed at 3000–3600 cm^−1^, which corresponds to the O–H groups of PVA and Na CMC in the physical mixture. This peak also appears in the spectrum of the CIP hydrogel patch at a lower intensity, where the OH groups of the polymers participate in hydrogen bond formation between PVA and CMC, as well as between the polymers and borax, to form the di-diol ester linkage. Additionally, the peak observed around 2900 cm^−1^ in both spectra corresponds to the C–H bonds that were originally present in the polymers. Moreover, the vibrational band observed between 1730 and 1740 cm^−1^ in both spectra corresponded to the acetate group remaining from PVA.

The FTIR spectrum of the CIP hydrogel patch exhibited peaks at 1419 and 1245 cm^−1^, corresponding to the characteristic B–O–R bonds (Figure 4B), indicating the formation of a diol borate ester linkage. The peak at 1105 cm^−1^ corresponds to the unreacted borate ions derived from borax [35,36]. A distinct peak at 677 cm^−1^ appeared in the spectrum of CIP hydrogel patch, but was absent in the physical mixture, indicating the bending of B–O–B junctions in borate ions. Additionally, the spectrum of the CIP hydrogel patch displayed a peak at 833 cm^−1^ that corresponds to the B–O bond vibration and a noticeable peak at 769 cm^−1^ that corresponds to the bending of C–H bonds formed in the CIP hydrogel patch [17]. Hence, the FTIR spectrum of the CIP hydrogel patch revealed the formation of new bonds, particularly with borax and borate ions (Table 1), suggesting the successful crosslinking of the CIP hydrogel patch.

### 3.4. Characterization of CIP Hydrogel Patches

#### 3.4.1. Drug Recovery

A calibration curve for CIP was prepared using a UV-vis spectrophotometer at λ_max_ of 270 nm to determine drug recovery from the CIP hydrogel patches. The linear equation of the CIP calibration curve was (y = 0.0928x + 0.0071) with a correlation coefficient (R^2^) = 0.9998 (Figure S3). The percentage of drug recovery from the CIP hydrogel patch was 94.41 ± 0.71%, complying with the USP standard, which requires the drug to be within 90–110% of the declared amount [37].

#### 3.4.2. Rheological Studies

Rheological studies evaluated the viscosity, linear viscoelastic region (LVR), and viscoelastic properties of the CIP hydrogel patches. These tests were conducted after 1, 24, and 48 h of crosslinking to assess the mechanical changes of the CIP hydrogel over time. Additionally, the self-healing ability of the CIP hydrogels was analyzed using the three-interval thixotropy test (3ITT).

##### Viscosity of CIP Hydrogels

**Figure 5:**
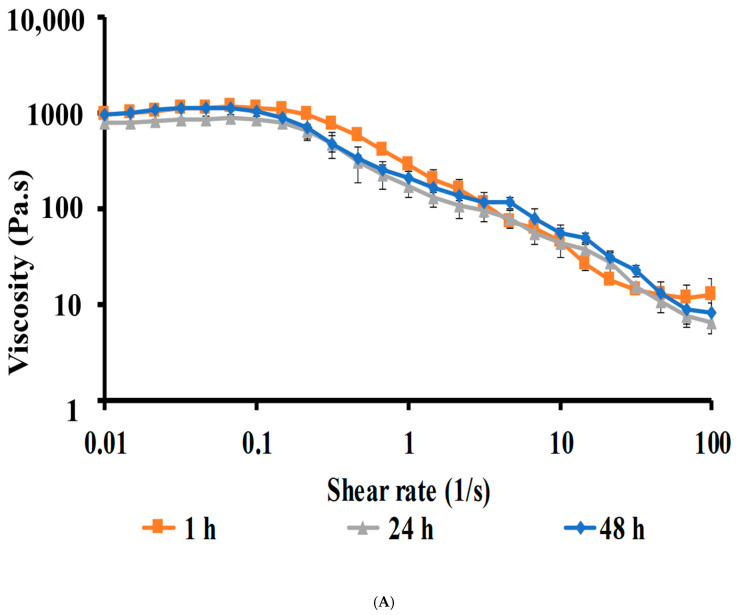

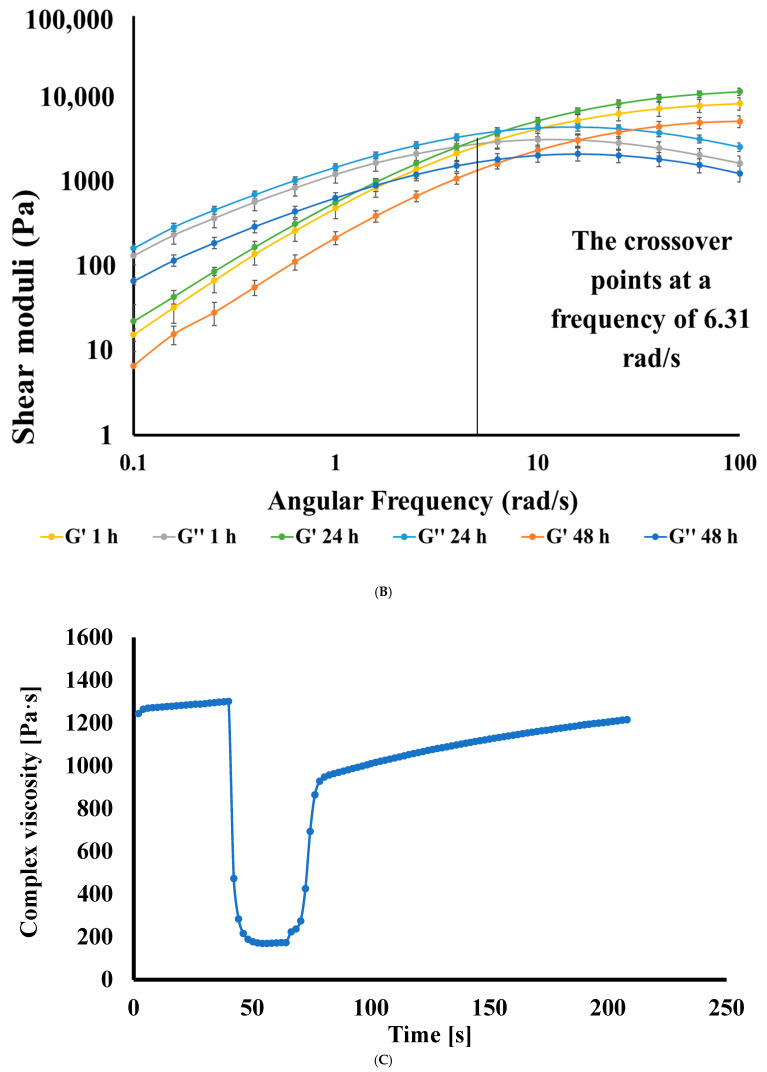
(**A**) Viscosity curves of CIP hydrogel patches crosslinked after 1, 24, and 48 h at 32 °C. (**B**) Frequency-dependent elastic modulus (G′) and viscous modulus (G″) demonstrate the crossover points (at 6.31 rad/s) for the CIP hydrogel patches crosslinked for 1, 24, and 48 h. (**C**) Three-interval thixotropy test revealed good self-healing ability of CIP hydrogel patch and recovery of 97.7 ± 2.5% in 2.4 min after the high shear strain was removed. Data are reported as mean ± SD (*n* = 3).

The CIP hydrogel patches exhibited pseudoplastic (shear-thinning) flow, characterized by a decrease in viscosity as the shear rate increases [25]. A plateau region was observed at low shear rates, indicating constant viscosity, where high crosslinking resists the applied stress [38]. Subsequently, a shear-thinning effect began at a shear rate of 0.1 s^−1^, resulting in induced flow. The shear-thinning behavior of self-healing hydrogels facilitates the injectability and 3D-printability of the hydrogel [39]. The dynamic covalent crosslinks enable the shear-thinning behavior during injection and self-healing after injection [39]. The viscosity curves of CIP hydrogel patches after 1, 24, and 48 h of crosslinking were comparable to one another (Figure 5A), indicating no change in the viscosity after 48 h.

##### Strain-Sweep Studies

The strain-sweep test was conducted to determine the linear viscoelastic region (LVR). This region refers to a strain range where the sample can be tested without compromising its internal structure when applying shear stress, and where G′ and G″ are independent of strain [40]. Therefore, establishing this region is essential because the hydrogel structure remains intact during the frequency-sweep tests [40]. The LVR extended from 0.01 to 2.51% for the CIP hydrogel patch after 1 h of crosslinking and shortened to 0.01–1.58% for CIP hydrogel patches after 24 and 48 h of crosslinking (Figure S4). The point at the end of the LVR where the hydrogel structure begins to break down is known as the critical strain (γ_C_). Beyond this point, a nonlinear material response of large-amplitude oscillatory shear was observed [41]. The LVRs, applied strain within the LVR, and γ_C_ are summarized in Table 2. A strain of 0.10%, which is within the LVR, was chosen for the subsequent frequency-sweep studies.

##### Frequency-Sweep Studies

The frequency sweep test was conducted to determine the viscoelastic properties (G′ and G′′) of the CIP hydrogel patches that were crosslinked for 1, 24, and 48 h over a frequency range of 0.1–100 rad/s (Figure 5B). The storage (elastic) modulus G′ represents the energy stored in a material, while the loss (viscous) modulus G″ signifies the energy loss [42]. For CIP hydrogel patches crosslinked for 1, 24, and 48 h, G″ dominated G′ at low frequencies, indicating a liquid-like behavior. Conversely, G′ surpassed G′′ at high frequencies, suggesting a solid-like behavior. This phenomenon occurs because energy loss arises from the breakage and reformation of physical crosslinks at low frequencies, when there is sufficient time for the bonds between the polymer chains to reform. In contrast, at high frequencies, the crosslink bonds lack the time needed to reform. As a result, the bonds store energy, and the hydrogel behaves as an elastic body [43].

The presence of crossover frequency points, where G′ = G″ at a frequency of 6.31 rad/s, indicates the existence of reversible crosslinking in the CIP hydrogel patches [38]. This crossover point remained consistent for CIP hydrogel patches crosslinked for 1, 24, and 48 h. At a frequency of 6.31 rad/s, the hydrogels demonstrated a transition from a liquid-like to a solid-like structure.

A noticeable change was observed in the viscoelastic properties of the CIP hydrogel patches crosslinked for 1, 24, and 48 h. After 24 h of crosslinking, more interactions between borax and the polymer chains formed compared to those established after 1 h of crosslinking, as evidenced by an increase in G′ and G″ of the CIP hydrogel patch. After 48 h of crosslinking, a decrease in G′ and G″ was noted. This might be attributed to relaxation in the network, where some of the weaker physical crosslinks and reversible diol-borate ester bonds might break or rearrange. Thus, the system might reach a new equilibrium where some of the crosslinks become weaker [44]. Therefore, unless otherwise specified, the CIP hydrogel patch crosslinked for 24 h was used for further characterization. Additionally, the hydrogel crosslinked for 24 h achieved the Petri dish shape.

##### Three-Interval Thixotropy Test (3ITT)

Thixotropy is a time-dependent and reversible change in the complex viscosity displayed by pseudoplastic systems. These systems exhibit a decrease in complex viscosity over time under constant shear, but can recover their initial complex viscosity after the shear is removed [45]. The three intervals of the thixotropy test provide insight into the degree of recovery following the deformation of the hydrogel (Figure 5C). The CIP hydrogel patch was subjected to a low shear strain of 0.1% during the first interval, chosen based on the LVR. Afterward, a high shear strain of 100% was applied in the second interval, and in the third interval, a shear strain of 0.1% was applied again. In the first interval, the complex viscosity was approximately 1200 Pa·s. When the high shear strain was applied, the complex viscosity dropped to about 160 Pa·s. In the third interval, after the removal of the high shear, the hydrogel bonds regenerated, and the complex viscosity roughly returned to its initial value of 1200 Pa·s. The CIP hydrogel patch recovered 97.7 ± 2.5% of its complex viscosity within 2.4 min after the high shear strain was removed, indicating that the CIP hydrogel patch has excellent self-healing ability.

#### 3.4.3. Morphology of CIP Hydrogel Patch

**Figure 6:**
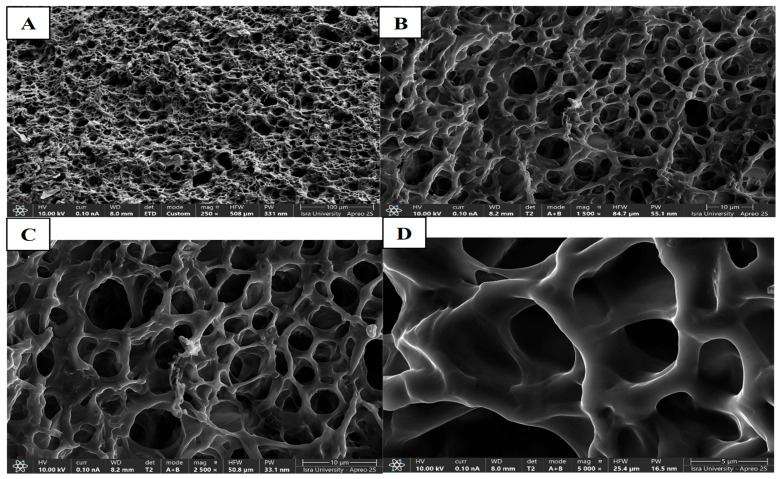
SEM images of CIP hydrogel patch observed under various magnifications: (**A**) 250×, (**B**) 1500×, (**C**) 2500×, and (**D**) 5000×.

The lyophilized CIP hydrogel patch was analyzed using a high-resolution field emission scanning electron microscope. Micrographs of the CIP hydrogel patch were collected at various magnifications (250×, 1500×, 2500×, and 5000×) (Figure 6). The 3D structure of the hydrogel revealed a highly porous and crosslinked matrix, indicating successful gel formation [23]. This porous structure plays a crucial role in determining the swelling capacity and swelling rate. High porosity increases the contact area of the polymer network with the surrounding aqueous solution, enhancing its ability to absorb and retain aqueous solutions [46].

#### 3.4.4. Swelling Behavior of CIP Hydrogel Patch

The swelling behavior is a characteristic property of hydrogels, which can absorb substantial water and retain it without dissolution [47]. The swelling behavior of the hydrogels was investigated to assess their ability to absorb wound exudate and maintain moisture in the wound region, thereby aiding in the wound healing process. Hydrogels, as a drug delivery system, can release drugs by swelling. When water is absorbed, the size of the matrix pores increases, allowing the drug to diffuse out. The CIP hydrogel patch showed a gradual increase in the swelling ratio, followed by a gradual decrease in weight due to erosion. The process of removing the hydrogel from the buffer solution at each time point, weighing it, and then returning it to the solution causes erosion of the hydrogel, leading to imprecise results. Therefore, the test was conducted at the 1 h time point, and each piece was weighed only once. The swelling percentage of the CIP hydrogel patch was 337.4 ± 12.7%.

#### 3.4.5. Self-Healing Ability

**Figure 7:**
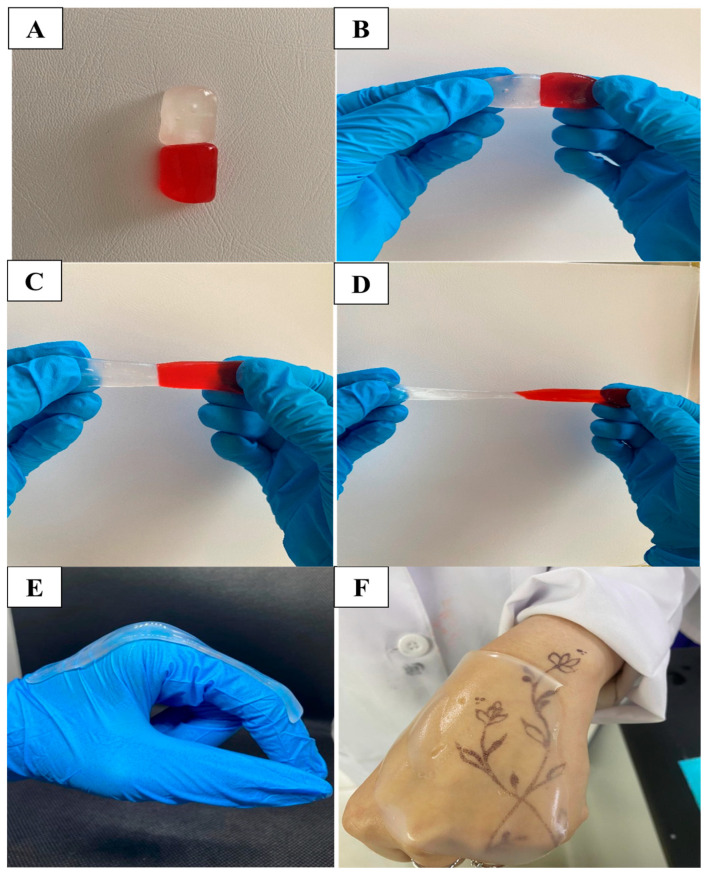
(**A**–**D**) Scheme illustrating the self-healing ability of CIP hydrogel patch without applying any external force, where it took 3–4 min for the hydrogel to regenerate its bonds. The healed hydrogel can be stretched without cracking or breaking at the healing site, and (**E**,**F**) photographs illustrate the CIP hydrogel patches applied to potential body sites that subject to movement human.

Self-healing hydrogel patches are suitable for wounds on the face and joints of the body, including elbows, knees, neck, ankles, and finger joints that are subject to movement, which can disrupt the integrity of the dressing and lead to secondary damage to the wound [48]. Self-healing hydrogels are suitable for use due to their stretchability, flexibility, and ability to self-repair if damaged [49]. To confirm the self-healing ability of the CIP hydrogel patch and its ability to recover after damage when subjected to movements while applied to the wound, a colored piece of the hydrogel was placed in contact with an uncolored piece and allowed to heal without any external forces at room temperature [23]. After they were attached for 3–4 min, the pieces merged into a single one, and the healed hydrogel could be stretched as one single piece without any cracks at the healing site (Figure 7A–D). Additionally, Figure 7E,F illustrate photographs of CIP hydrogel patches applied to human joints.

#### 3.4.6. Stability Studies of CIP Hydrogel Patch

The physical stability of the CIP hydrogel patches, including appearance, transparency, and drug precipitation, was evaluated after storage for one and three months at room temperature. The CIP hydrogel patches showed no drug precipitation. However, a few patches showed signs of bacterial and fungal growth after three months (Figure S5).

After one month of storage at room temperature, the self-healing ability test revealed that the hydrogel retains its healing ability, taking approximately 3 min to heal completely (Figure S6). Additionally, the CIP hydrogel patches retain their ability to stretch 35 times their original length after one month (Figure S7), with 93.04 ± 2.85% of the drug being recovered after this period. However, a three-month stability study was not conducted due to the microbial contamination of the patches. This observed microbial contamination might be due to the exposure of CIP hydrogel patches to the environment, as the patches were not covered or placed in sealed containers, unlike the commercially available patches that are sealed and not exposed.

**Figure 8:**
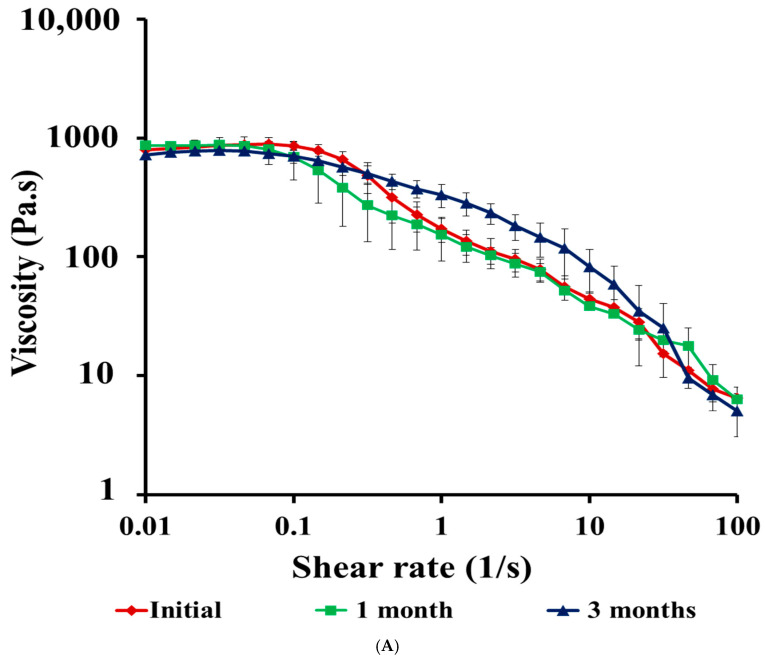

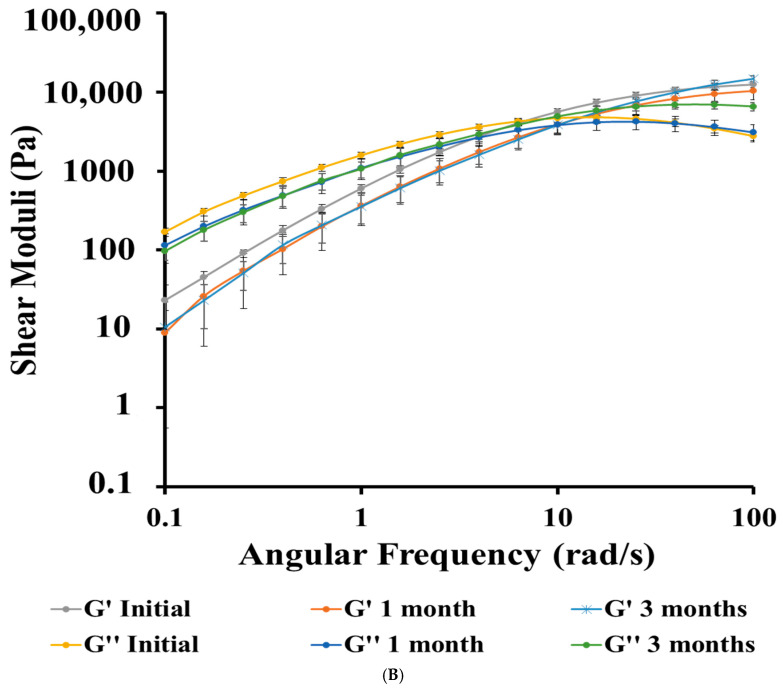
(**A**) Viscosity curves of the CIP hydrogel patches and (**B**) The elastic modulus (G′) and loss modulus (G″) of the CIP hydrogel patches after one month and 3 months of storage at room temperature. The viscosity and viscoelastic properties of the CIP hydrogel patch crosslinked after 24 h was added to the figures for comparison. Data are presented as the mean ± SD (*n* = 3).

Rheological stability studies were performed on the CIP hydrogel patches after one and three months of storage at room temperature, and the results were compared with those of their respective initial rheological measurements. The viscosity curves indicated that the CIP hydrogel patches maintained the plateau region at low shear rates, followed by pseudoplastic flow behavior after one and three months of storage (Figure 8A). There was no significant difference between the viscosity results of stored CIP hydrogel patches and those obtained after 24 h of crosslinking, as confirmed by the one-way ANOVA (*p* = ns). The LVR for the CIP hydrogel patches was maintained after one month of storage (0.01–1.58%) and decreased to 0.01–1.00% after three months (Table 2, Figure S8), indicating that the hydrogel underwent structural changes.

After one and three months of storage, the viscoelastic properties (G′ and G″) of the CIP hydrogel patches were maintained; however, a decrease in G′ was observed during storage (Figure 8B). The crossover point for the initial hydrogel, crosslinked for 24 h, shifted from 6.31 rad/s (Figure S9A) to a higher frequency of 10 rad/s after one month (Figure S9B), followed by a further increase in the crossover point after 3 months, reaching 15.8 rad/s (Figure S9C). The crossover point is related to the relaxation time of the hydrogel. As the crossover point shifts to a higher frequency, the relaxation time of the hydrogel decreases [50,51]. Therefore, the shift of the crossover point to higher frequencies implies that the hydrogel responds faster to the applied stress over time. This indicates that the hydrogel structure is weaker after one and three months, as the bonds break down, making the hydrogel more liquid-like over a wide range of frequencies before it converts to a solid-like structure [51].

### 3.5. Antimicrobial Study

#### 3.5.1. Well-Diffusion Test

**Figure 9:**
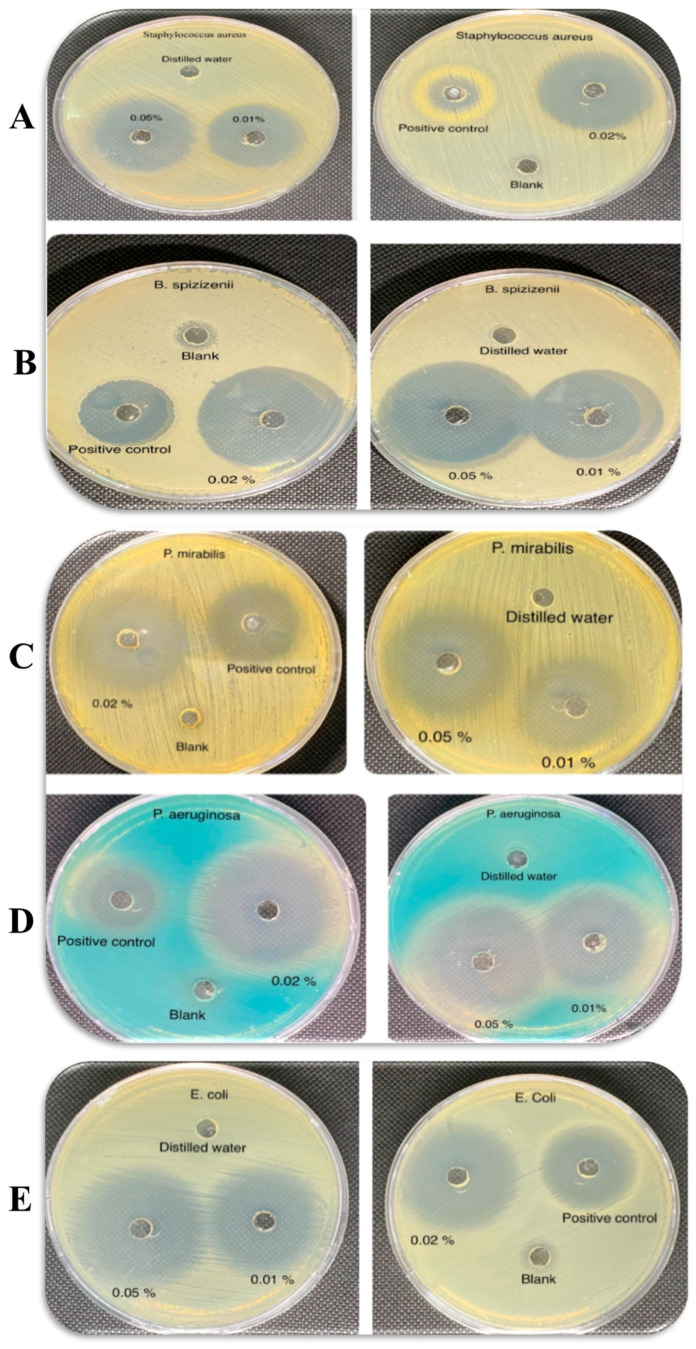
The antimicrobial activity of CIP hydrogel patches (0.01, 0.02, and 0.05%), positive control, and negative control against (**A**) *S. aureus*, (**B**) *B. spizizenii*, (**C**) *P. mirabilis*, (**D**) *P. aeruginosa*, and (**E**) *E. coli*.

Wounds are highly susceptible to colonization by various types of bacteria, which can delay the healing process. The antimicrobial activity of CIP hydrogel patch was evaluated by measuring the inhibitory zone against three Gram-negative bacteria (*E. coli* ATCC8739, *P. aeruginosa* ATCC9027, and *P. mirabilis* ATCC12453) and two Gram-positive bacteria (*B. spizizenii* ATCC6633 and *S. aureus* ATCC6538). The wells were filled with 50 μL of distilled water (negative control), 5 μg/mL CIP solution (positive control), and pieces of CIP hydrogel patches with a diameter of 6 mm, with CIP concentrations ranging from 0.01% to 0.05%. Zones of inhibition were visible for the positive control and three concentrations of CIP hydrogel patches (0.01, 0.02, and 0.05%) tested against both Gram-positive and Gram-negative bacteria, indicating that CIP effectively diffused from the hydrogel matrix (Figure 9).

By comparing the zone of inhibition, the antimicrobial activity of CIP hydrogel patches showed no significant differences between Gram-positive and Gram-negative bacteria (Table 3). The CIP hydrogel patch containing 0.05% CIP exhibited the highest inhibitory effect, representing the most stable concentration, which was used in further characterization.

#### 3.5.2. Minimum Inhibitory Concentration

Both the broth dilution and agar-diffusion methods are used to determine the minimum inhibitory concentration (MIC), with the first method providing a quantitative analysis and the second offering a semi-quantitative assessment [52,53]. The well-diffusion method evaluates the ability of hydrogels to release the drug from the matrix, inhibiting bacterial growth. Hence, it more accurately reflects the actual drug release from the formulation and provides a better measurement of the MIC within the formulation. For example, the agar well diffusion method was employed to evaluate the antibacterial activity of triple antibiotics (ciprofloxacin, metronidazole, and minocycline) and (ciprofloxacin, metronidazole, and amoxicillin) in paste and hydrogel forms against *Enterococcus faecalis* [54].

A concentration of 5 µg/mL of CIP has been used as a positive control by Singaravelu et al. [55]. Higher concentrations of CIP in CIP hydrogel patches were used to assess their antimicrobial activity, considering the potential delay in drug release from the formulation and/or any hindrance to drug release. The MIC is the lowest concentration of CIP at which a visible inhibition zone can be observed. Fifteen concentrations of CIP in the hydrogels (570, 250, 110, 50, 28, 14, 7, 3, 1.7, 0.85, 0.4, 0.2, 0.1, 0.05, and 0.02 μg/mL) were tested (Table 3). By comparing the antimicrobial activity represented by the zone of inhibition of the positive control (5 μg/mL) with those of CIP hydrogel patches (7 and 3 μg/mL), it was found that the antimicrobial activity of the positive control against the bacterial species was nearly equal to or higher than that of the hydrogels. This is because the positive control exists in a solution state; hence, no diffusion is required. *E. coli* has the lowest MIC (0.05 μg/mL) among the tested species, followed by *B. spizizenii* (0.2 μg/mL). The *S. aureus* and *P. aeruginosa* each have an MIC of 1.7 μg/mL, while *P. mirabilis* has an MIC of 0.85 μg/mL.

Masadeh et al. [56] reported zones of inhibition measuring 26.7, 19.7, 23.3, and 22.7 μg/mL against *E. coli*, *P. mirabilis*, *P. aeruginosa*, and *S. aureus*, respectively, using a 100 μg/mL CIP solution. Similarly, Akhlaq et al. [57] prepared a serial dilution of CIP solutions (ranging from 1000 to 1.95 µg/mL) in Mueller–Hinton broth and observed inhibition zones of 14 and 12 mm against *S. aureus* and *E. coli*, respectively, at a concentration of 1000 µg/mL. In comparison, the CIP hydrogel patches developed in the present study demonstrated larger zones of inhibition, suggesting superior antimicrobial efficacy. However, a subsequent investigation by Masadeh et al. [58] reported that the MIC of CIP solutions was lower than that of our CIP hydrogel patch. This discrepancy might be attributed to the limited sensitivity of the agar diffusion method in accurately determining the MIC values [52], as well as the relatively low concentration of CIP in the hydrogel, which may hinder its effective diffusion from the hydrogel matrix.

### 3.6. In Vitro Cytotoxicity Study

**Figure 10:**
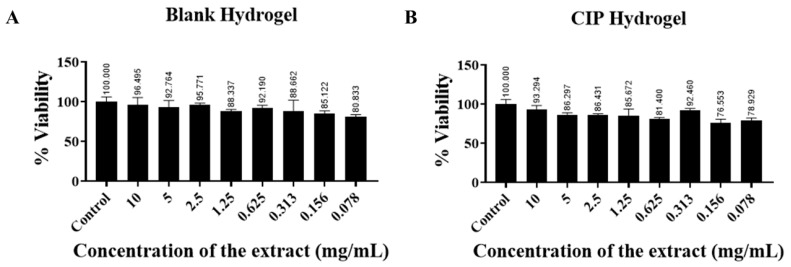
Indirect cytotoxicity for the extracts obtained from (**A**) blank hydrogel patch and (**B**) CIP hydrogel patch on the HDFa cell line. Data are presented as the mean ± SD (*n* = 3).

There was no significant reduction in cell viability using the blank hydrogel and CIP hydrogel patches, even at high extract concentration (10 mg/mL). According to ISO standards, a material is considered non-cytotoxic if cell viability is greater than 70% after exposure to the material [59]. The results showed that the cell viability was greater than 70% for both the blank hydrogel and the CIP hydrogel patches (Figure 10). Hence, the CIP hydrogel patches are considered biocompatible and safe for use in further animal studies, with good potential for clinical application.

### 3.7. Bioadhesion of CIP Hydrogels

It is essential for the hydrogels to possess a bioadhesive property to ensure prolonged residence time and release the active substance at the site of application [60]. The abundance of hydroxyl groups in the two polymers (PVA and Na CMC) endows the hydrogel with a bioadhesive property, enabling it to form bonds with various functional groups on the skin surface.

**Figure 11:**
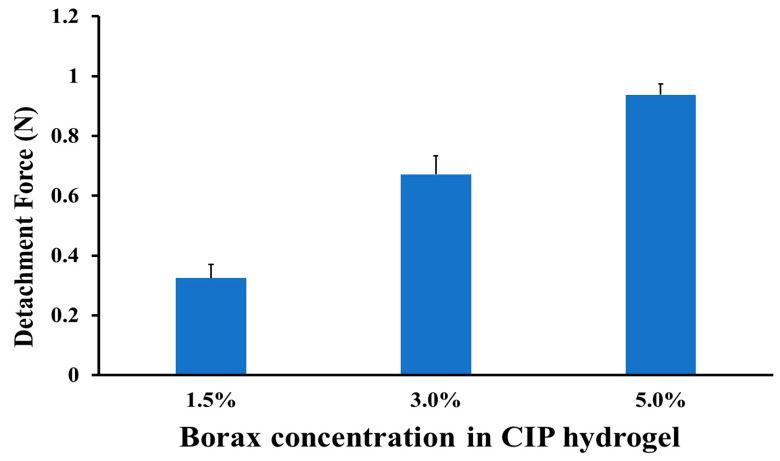
Detachment force between the rat skin and the CIP hydrogels represents the effect of borax concentration (1.5, 3.0, 5.0%) on the detachment force. Data are presented as mean ± SD (*n* = 3).

The bioadhesive property of the CIP hydrogel was assessed to evaluate the effect of borax concentration on the detachment force between the skin and the CIP hydrogel. The detachment force is the force required to overcome the adhesive bonds between the hydrogel and the skin [61]. The results showed that increasing the borax concentration increased bioadhesion strength, as indicated by the enhancement in the detachment force (Figure 11), in agreement with Chen et al. [62]. The detachment forces for the CIP hydrogels crosslinked with 1.5, 3, and 5% borax were 0.325 ± 0.04, 0.671 ± 0.06, and 0.937 ± 0.03 N, respectively. This might be due to the liquid-like nature of the CIP hydrogel crosslinked with a low borax concentration (1.5%) compared to those crosslinked with higher borax concentrations (3 and 5%). Additionally, it was noticed that as the borax concentration increases, the crosslinking density increases until the highest crosslinking density is achieved [43]. Thus, with increasing borax concentration, more crosslinks are formed, and the bioadhesive strength of the hydrogel increases. Hence, the good cohesion of the hydrogel contributes to its good bioadhesion property, which is attributed to its good mechanical stability [63,64]. Although the results of Chen et al. [62] were consistent with our findings, where increasing borax concentration increases the adhesiveness of starch-borax hydrogels, other studies reported that increasing borax concentration leads to a decrease in the adhesiveness of PVA-borax hydrogels [65,66].

Although the hydrogel formed with a 5% borax concentration resulted in higher bioadhesive strength, the 3% borax concentration was chosen for further studies. This is because the CIP hydrogel crosslinked with 3% borax demonstrated good bioadhesion and mechanical properties, as it relaxed and filled the Petri dish after 24 h, unlike the hydrogel formed with 5% borax concentration (Figure 2).

### 3.8. Ex Vivo Permeation

**Figure 12:**
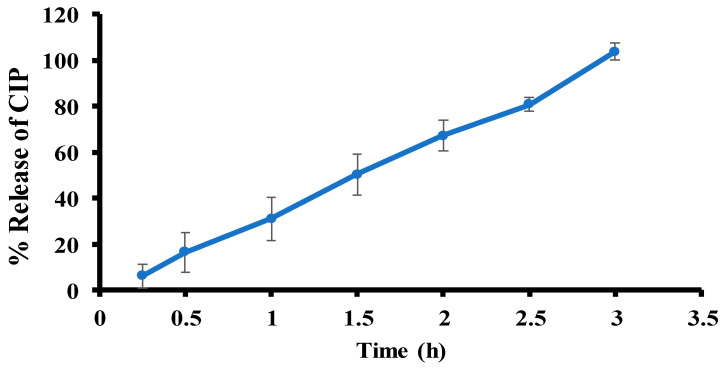
Ex vivo permeation profile of the CIP hydrogel patch. Data are presented as the mean ± SD (*n* = 3).

The ex vivo permeation study was performed using a UV-vis spectrophotometer. A calibration curve for CIP was established using the linear equation y = 0.0928x + 0.0071 with a correlation coefficient (R^2^) of 0.9998. The ex vivo permeation profile of CIP from the optimal CIP hydrogel patch showed a burst-release pattern. The percent release of CIP (Q%) from the hydrogel was plotted against time (Figure 12). After 3 h, the cumulative amount of CIP permeated per unit surface area of rat skin was 215.8 ± 20.2 µg/cm^2^, corresponding to 103.7 ± 3.7%. Additionally, the associated steady-state flux (Jss) was 72.9 ± 7.7 µg/cm^2^/h, and the permeability (P) was 61.02 ± 2.88 × 10^−2^ cm/hr. The high release rate of CIP from the hydrogel is likely due to its porous structure, which allows it to swell upon contact with PBS. The small molecular weight of the drug allows it to diffuse from the hydrogel matrix as the hydrogel swells, thereby increasing the pore size.

It has been shown that controlled release of wound healing patches would be beneficial for maintaining a constant drug concentration over time and reducing the need for frequent dressing changes [67]. However, the burst release of the CIP hydrogel patch involves delivering drugs at high drug rates at the beginning of wound treatment to combat infection and reduce biofilm formation [68,69].

Additionally, the local release of CIP from CIP hydrogel patches might contribute to bacterial resistance [70]. Therefore, new approaches have been explored to reduce bacterial resistance against CIP. These include incorporating nitric oxide and silver nanoparticles into CIP hydrogel patches [71] and combining CIP with other antibacterial agents from various classes, natural products, bacteriophages (viruses that infect bacteria), and photodynamic therapy [70]. This could enhance the efficacy of CIP hydrogel patches and increase their bacterial sensitivity, thereby helping to prevent the development of resistance.

### 3.9. In Vivo Wound Healing Study—A Pilot Study

As proof of concept for the wound-healing effect of CIP hydrogel, a pilot in vivo wound healing study was conducted with two mice per group. This experiment evaluated the degree of wound healing by applying CIP hydrogel and blank hydrogel patches five times a week until the wounds had healed, which took about 9 days. The results revealed that mice treated with the CIP hydrogel patch (Group 2) exhibited greater healing potential compared to the other two groups (Groups 1 and 3) (Table 4). Mice treated with the blank hydrogel patch showed that most wounds had healed, whereas the negative control mice showed dry wounds by the end of the experiment (after 9 days). This could be attributed to the ability of the hydrogel (both the blank and the CIP hydrogel patches) to maintain a moist wound bed. It has been proven that a moist environment encourages the healing process by preventing dehydration, enhancing angiogenesis and collagen synthesis, reducing pain, and inhibiting scar formation [72].

## 4. Conclusions

The optimal medicated CIP hydrogel patches, composed of 0.05% CIP, 15% PVA, and 2.5% Na CMC, and crosslinked with 3% borax, were successfully developed as wound dressings with the desired mechanical properties. The CIP hydrogel patch demonstrated self-healing and adhesive properties, rendering it suitable for wound healing at various locations while enhancing the healing process by ensuring prolonged residence time and complete drug release. The microstructural analysis of the CIP hydrogel patch revealed an interconnected porous matrix that supports other findings, such as swelling and retaining water within the matrix. Additionally, this study supports the hypothesis that the hydrogel was successfully crosslinked, as confirmed by the FTIR analysis. The self-healing ability of the CIP hydrogel patch was visually tested by merging pieces of different colors within a short time. Furthermore, rheological analysis confirmed the self-healing ability through the three-interval thixotropy test, which reveals that the CIP hydrogel patch can reconstruct its bonds after high shear application. The CIP content in the hydrogel was examined and found to be high. The antimicrobial analysis indicated that the CIP hydrogel patch is effective in eradicating bacteria. The cytotoxicity study showed that the CIP hydrogel patch is biocompatible and safe. The in vivo study on mice revealed promising findings, showing that wounds treated with both blank and CIP hydrogel patches had better outcomes compared with the negative control group due to the moist environment, which prevents wound dryness and scar formation.

However, a few limitations were present in this study, which warrant further investigation in future studies. For instance, the release properties of the CIP hydrogel patch could be modified to provide dual release kinetics, burst and sustained release, by exploring different combinations of PVA, Na CMC, and the crosslinker borax. Additionally, to minimize contamination, CIP hydrogel patches should be prepared in a controlled, cleanroom environment or initially sterilized using UV radiation, and then placed in sealed packages before conducting a stability study. Finally, for broader validation, in vivo wound healing studies should be performed on additional wound models, such as splinting the wound in mice and the rat tail model [73,74], to better mimic human wound healing, along with extended monitoring periods. Furthermore, comparing the wound-healing effects of the CIP hydrogel patch with standard hydrogel dressings or commercially available antibiotic patches is crucial to better demonstrate its clinical potential.

## Figures and Tables

**Table 1:** Characteristic peaks in the FTIR spectrum of the CIP hydrogel patch.

Wavelength (cm^−1^)	Functional Group
1419 and 1245	B–O–R
677	B–O–B (bending)
769	C–H (bending)
833	B–O (vibration)

**Table 2:** Rheological parameters of the amplitude-sweep test of CIP hydrogel patches crosslinked with 3% borax for 1, 24, and 48 h.

Time of Crosslinking (h)	LVR (%)	Applied Strain Within the LVR (%)	Critical Strain (γ_C_, %)
1 h	0.01–2.51	0.1	2.51
24 h	0.01–1.58	0.1	1.58
48 h	0.01–1.58	0.1	1.58
1 month	0.01–1.58	0.1	1.58
3 months	0.01–1.00	0.1	1.00

**Table 3:** Zones of inhibition of CIP hydrogel patches of various concentrations, positive control of CIP solution of 5 g/mL, and negative control of distilled water using the agar well diffusion method. Results are displayed as mean diameters (mm) ± SD (*n* = 3).

		Zones of Inhibition (mm)				
		Gram-Positive Bacteria		Gram-Negative Bacteria		
Tested Samples	Concentrations (μg/mL)	*S. aureus*	*B. spizizenii*	*P. aeruginosa*	*P. mirabilis*	*E. coli*
D.W (negative control)	-	NZ	NZ	NZ	NZ	NZ
Blank hydrogel patch (negative control)	-	NZ	NZ	NZ	NZ	NZ
CIP solution (positive control)	5	16.3 ± 1.1	24.0 ± 0.0	17.8 ± 1.7	27.0 ± 0.0	24.6 ± 0.5
CIP hydrogel patch	570	33.3 ± 0.5	39.2 ± 0.3	36.3 ± 0.2	33.0 ± 0.0	33.6 ± 0.5
CIP hydrogel patch	250	31.0 ± 0.0	38.2 ± 0.3	34.1 ± 1.2	31.1 ± 0.2	30.6 ± 0.5
CIP hydrogel patch	110	29.3 ± 0.5	35.0 ± 0.0	31.0 ± 0.0	28.0 ± 0.0	29.8 ± 1.2
CIP hydrogel patch	50	26.3 ± 0.5	34.0 ± 1.4	28.6 ± 0.5	26.0 ± 0.2	27.3 ± 0.5
CIP hydrogel patch	28	24.0 ± 0.0	31.5 ± 0.7	25.6 ± 0.5	24.6 ± 0.5	25.3 ± 0.5
CIP hydrogel patch	14	19.6 ± 0.5	29.0 ± 0.0	24.0 ± 0.0	23.3 ± 0.5	23.8 ± 1.0
CIP hydrogel patch	7	17.3 ± 0.5	26.5 ± 0.7	21.0 ± 0.5	23.6 ± 0.5	22.6 ± 0.5
CIP hydrogel patch	3	13.6 ± 0.5	23.2 ± 0.3	17.3 ± 0.5	22.6 ± 0.5	21.3 ± 0.5
CIP hydrogel patch	1.7	10.0 ± 1.0	20.5 ± 0.7	13.1 ± 1.0	22.0 ± 0.0	20.3 ± 0.5
CIP hydrogel patch	0.85	NZ	18.7 ± 0.3	NZ	19.6 ± 0.5	19.5 ± 0.5
CIP hydrogel patch	0.4	NZ	14.0 ± 0	NZ	NZ	18.6 ± 0.5
CIP hydrogel patch	0.2	NZ	10.7 ± 1.0	NZ	NZ	15.1 ± 0.2
CIP hydrogel patch	0.1	NZ	NZ	NZ	NZ	12.8 ± 0.2
CIP hydrogel patch	0.05	NZ	NZ	NZ	NZ	9.8 ± 0.2
CIP hydrogel patch	0.02	NZ	NZ	NZ	NZ	NZ

NZ: represents no inhibitory zone.

**Table 4:** Photographs representing the in vivo wound healing study on mice using a negative control, CIP hydrogel patch, and blank hydrogel patch.

Groups	Day 1	Day 4	Day 9
Negative control (Group 1)	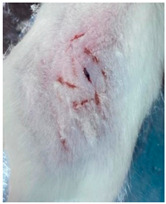	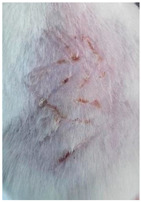	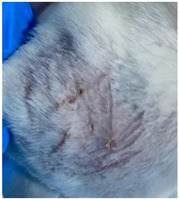
CIP hydrogel patch (Group 2)	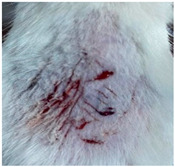	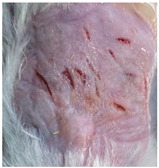	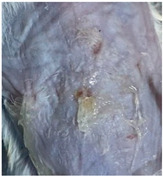
Blank hydrogel patch (Group 3)	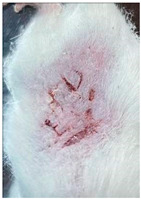	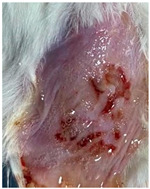	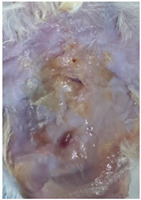

## Data Availability

Data available from the requests to the corresponding author.

## Supplementary Materials

The following supporting information can be downloaded at: https://www.mdpi.com/article/10.3390/polym17192686/s1, Figure S1: A scheme depicting the preparation method of the CIP hydrogel patch; Figure S2: Bioadhesion test setup, where the skin was fixed to the texture analyzer probe, and the CIP hydrogel patch was positioned on a cylindrical plastic support; Figure S3: The calibration curve of CIP prepared using a UV-Vis spectroscopic at λ_max_ of 270 nm at a concentration range of (0.15–10 μg/mL); Figure S4: The LVRs of CIP hydrogel patches crosslinked for (**A**) 1 h, (**B**) 24 h, and (**C**) 48 h. Data are reported as mean ± SD (*n* = 3); Figure S5: Three-month stability study of CIP hydrogel patches, where a few patches showed bacterial or fungal growth; Figure S6: The hydrogel maintains its self-healing ability after one month of storage at room temperature; Figure S7: The CIP hydrogel patch maintains its ability to stretch after one month of storage at room temperature; Figure S8: The LVRs of CIP hydrogel patches crosslinked for (**A**) 1 month and (**B**) 3 months. Data are reported as mean ± SD (*n* = 3); Figure S9: Crossover frequency points of CIP hydrogel patch after (**A**) one month and (**B**) three months of storage at room temperature. Data are reported as mean ± SD (*n* = 3).
