# Supplementary material for: Development, Characterization, and Biological Evaluation of a Self-Healing Hydrogel Patch Loaded with Ciprofloxacin for Wound Dressings
Source: Polymers (Basel). 2025 Oct 4;17(19):2686. doi: 10.3390/polym17192686 (PMC12527037; doi:10.3390/polym17192686)
Supplement: Supplementary file 1 [file polymers-17-02686-s001.zip › polymers-3850007-supplementary.pdf]

Supplementary File

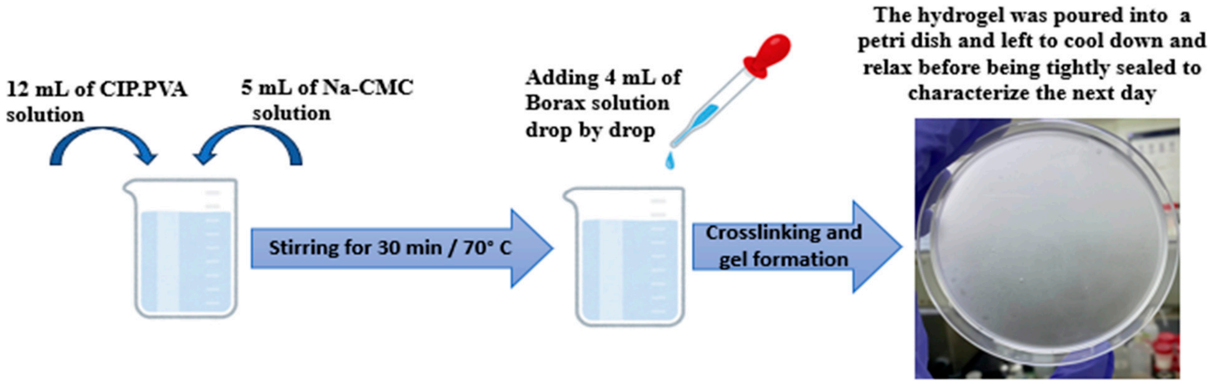

Figure S1: A scheme depicting the preparation method of the CIP hydrogel patch.

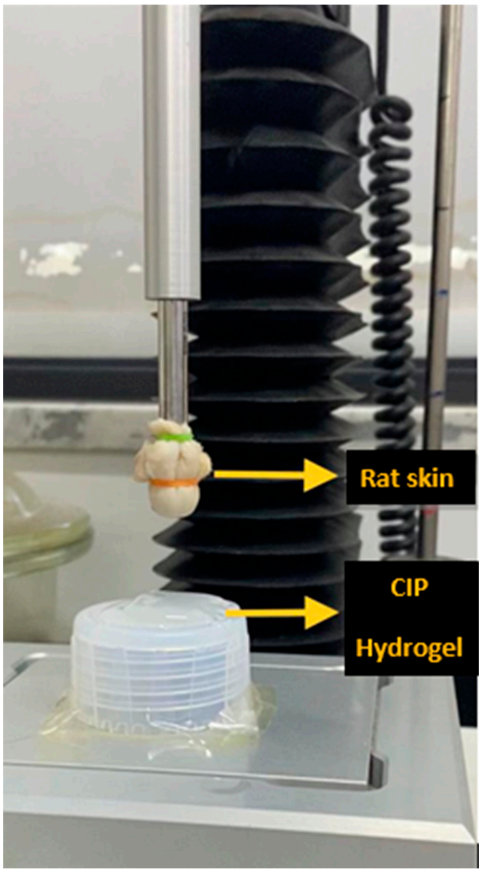

Figure S2. Bioadhesion test setup, where the skin was fixed to the texture analyzer probe, and the CIP hydrogel patch was positioned on a cylindrical plastic support.

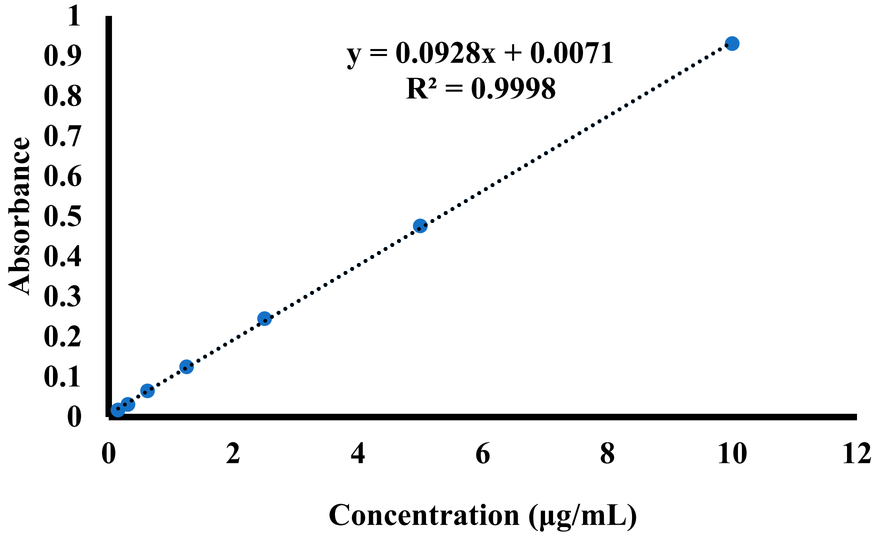

Figure S3: The calibration curve of CIP prepared using a UV-Vis spectroscopic at λ_{max} of 270 nm at a concentration range of (0.15-10 μg/mL).

A)

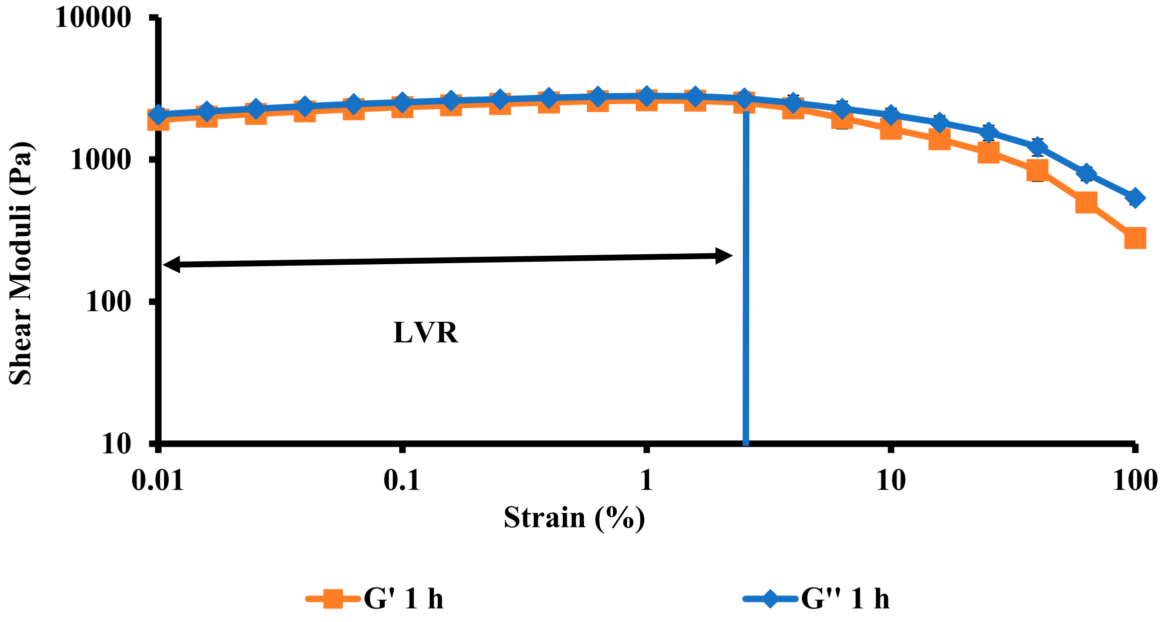
10000
1000
100
10
Shear Moduli (Pa)
LVR
0.01
0.1
1
10
100
Strain (%)
G' 1 h
G'' 1 h

B)

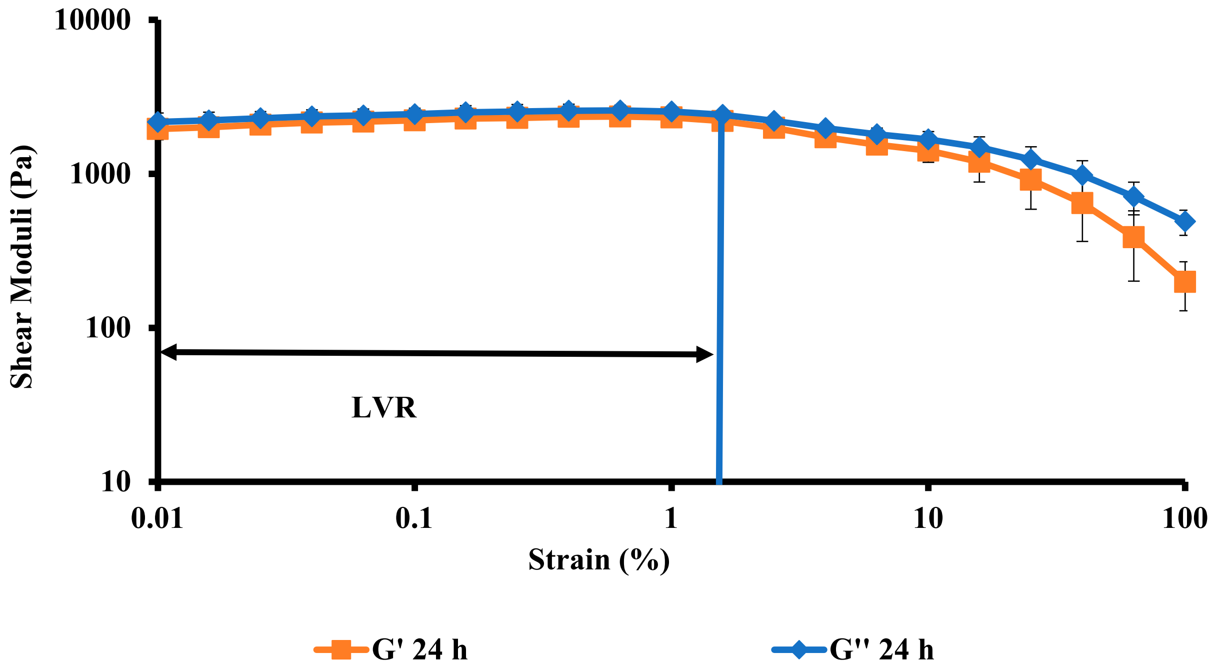
10000
1000
100
10
Shear Moduli (Pa)
LVR
0.01
0.1
1
10
100
Strain (%)
G' 24 h
G'' 24 h

C)

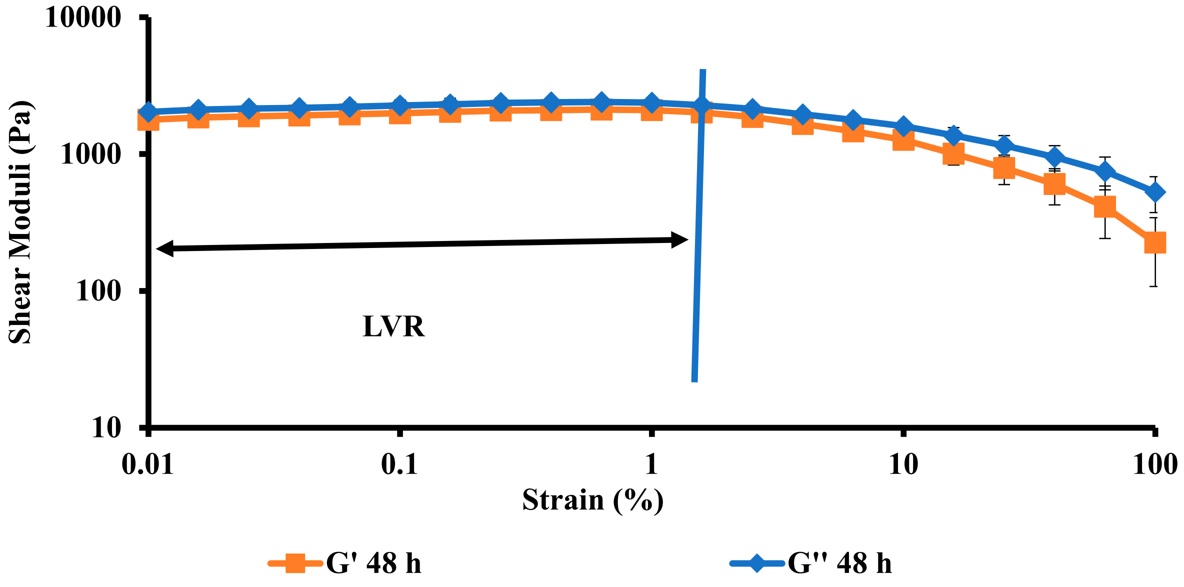

Figure S4. The LVRs of CIP hydrogel patches crosslinked for **(A)** 1 h, **(B)** 24 h, and **(C)** 48 h. Data are reported as mean ± SD (n=3).

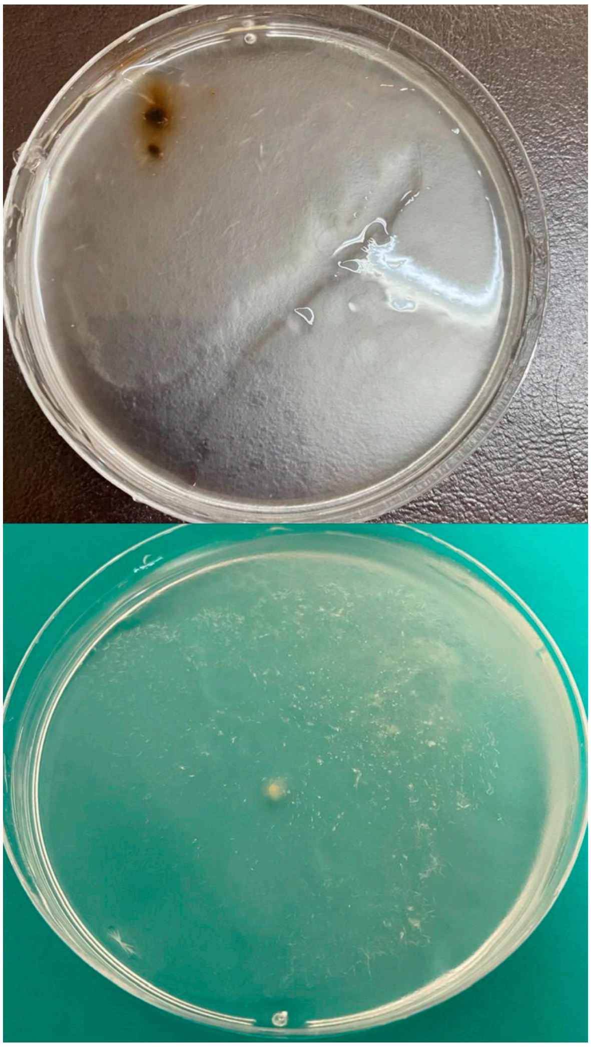

Figure S5: Three-month stability study of CIP hydrogel patches, where a few patches showed bacterial or fungal growth.

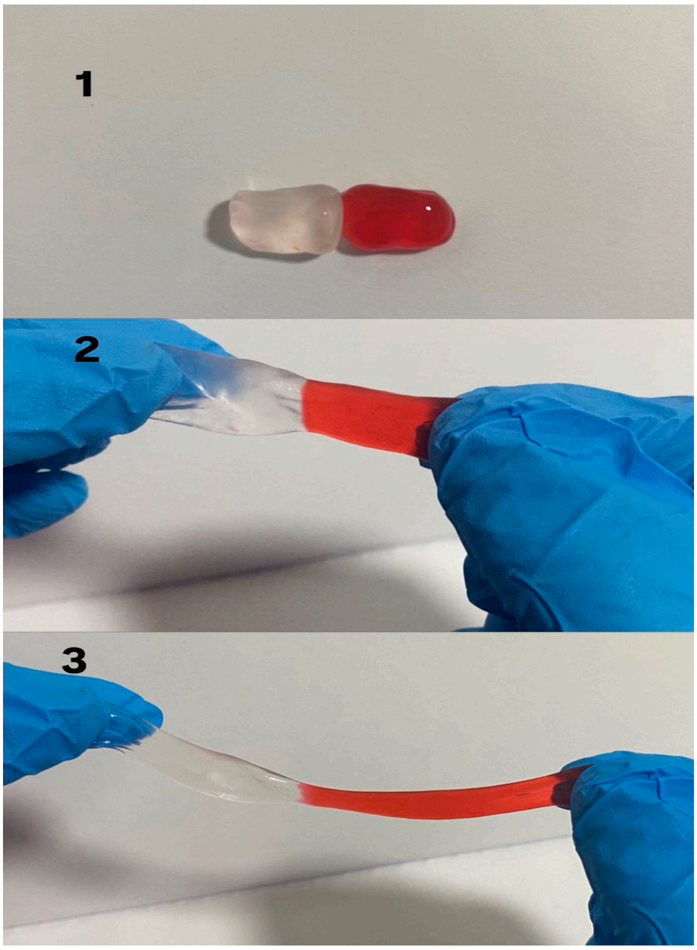

Figure S6: The hydrogel maintains its self-healing ability after one month of storage at room temperature.

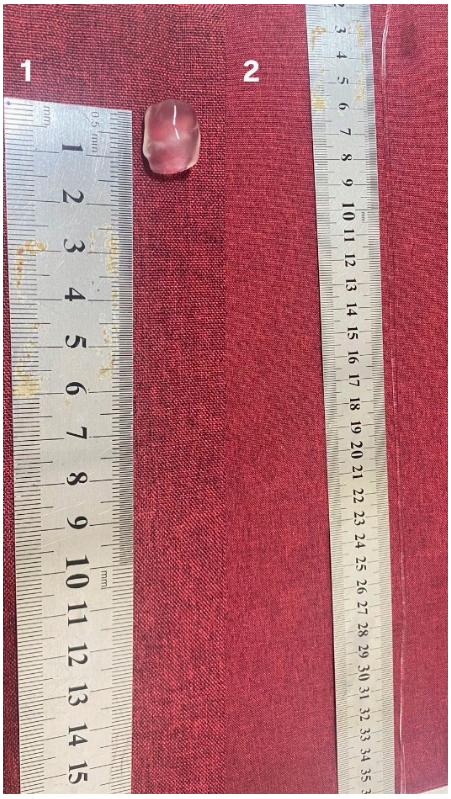

Figure S7: The CIP hydrogel patch maintains its ability to stretch after one month of storage at room temperature.

A)

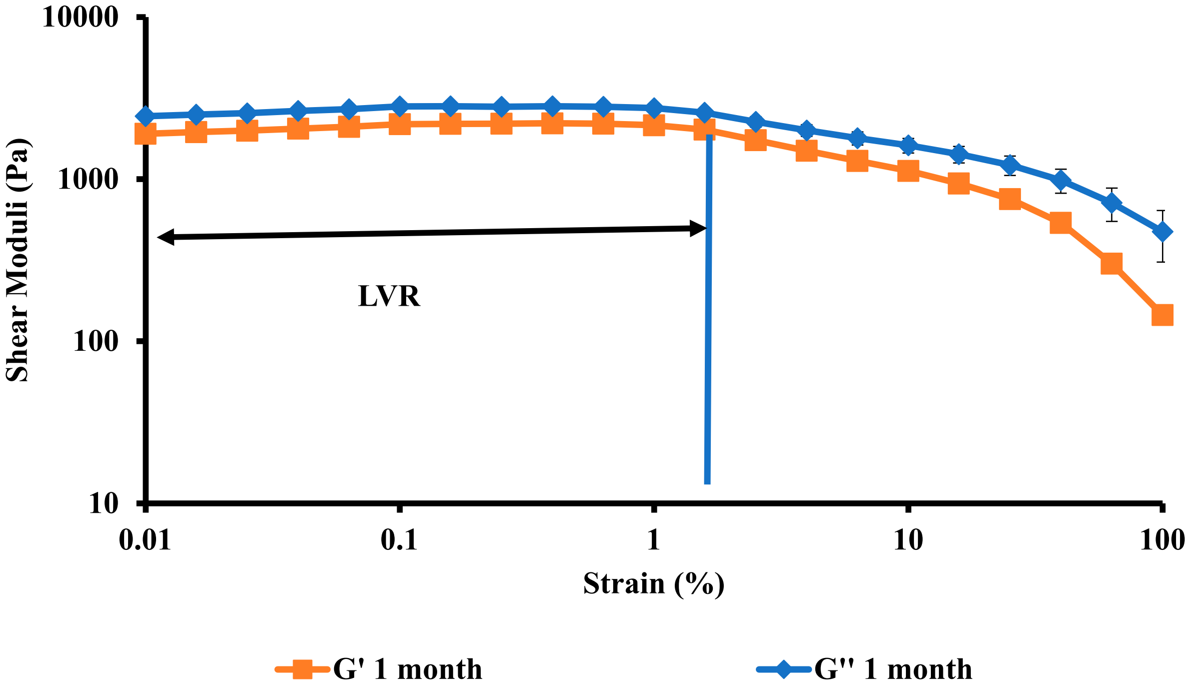
10000
1000
100
10
Shear Moduli (Pa)
LVR
0.01
0.1
1
10
100
Strain (%)
G' 1 month
G'' 1 month

B)

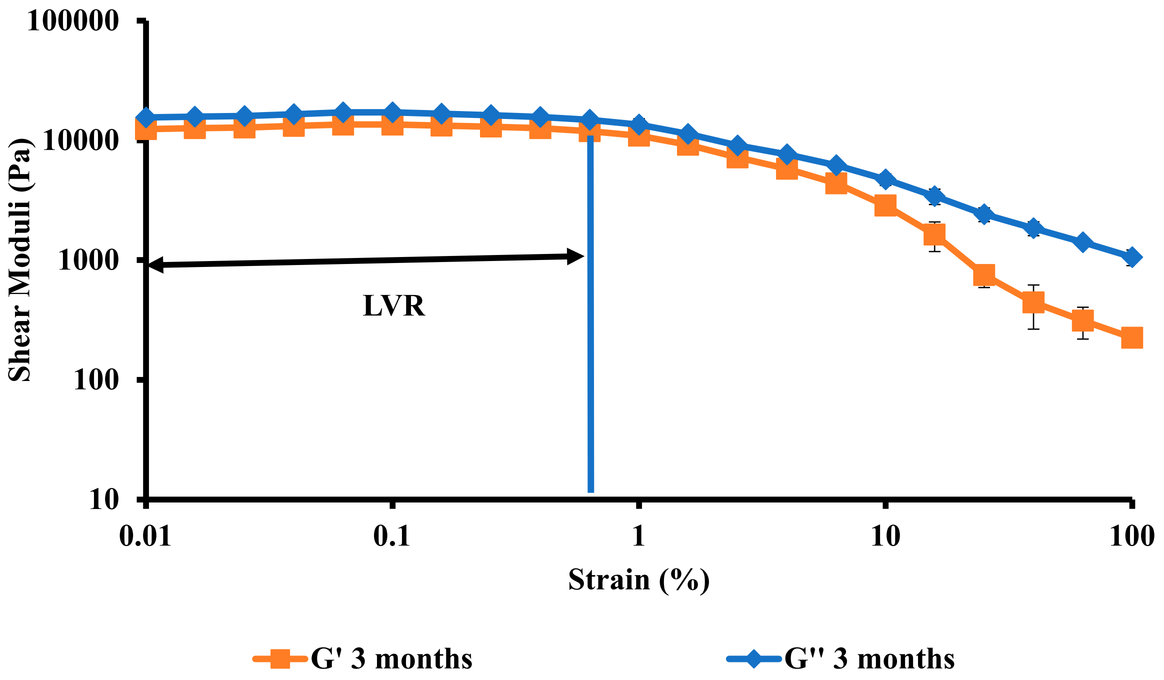

Figure S8. The LVRs of CIP hydrogel patches crosslinked for **(A)** 1 month and **(B)** 3 months. Data are reported as mean ± SD (n=3).

A)

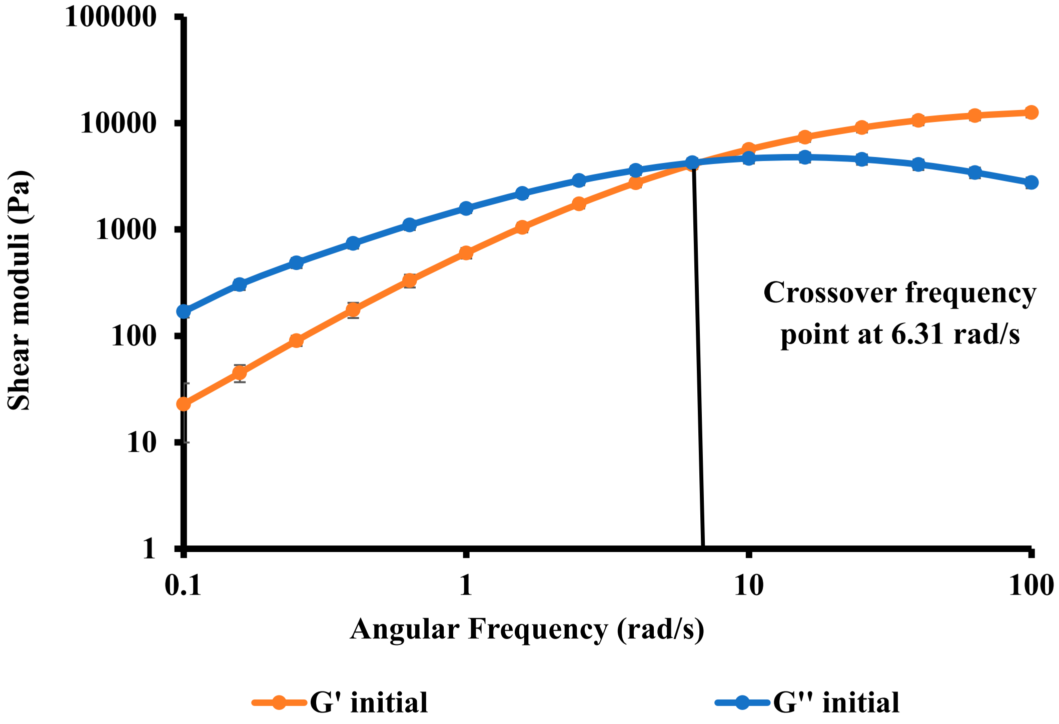
100000
10000
1000
100
10
1
Shear moduli (Pa)
0.1
1
10
100
Angular Frequency (rad/s)
Crossover frequency
point at 6.31 rad/s
G' initial
G'' initial

B)

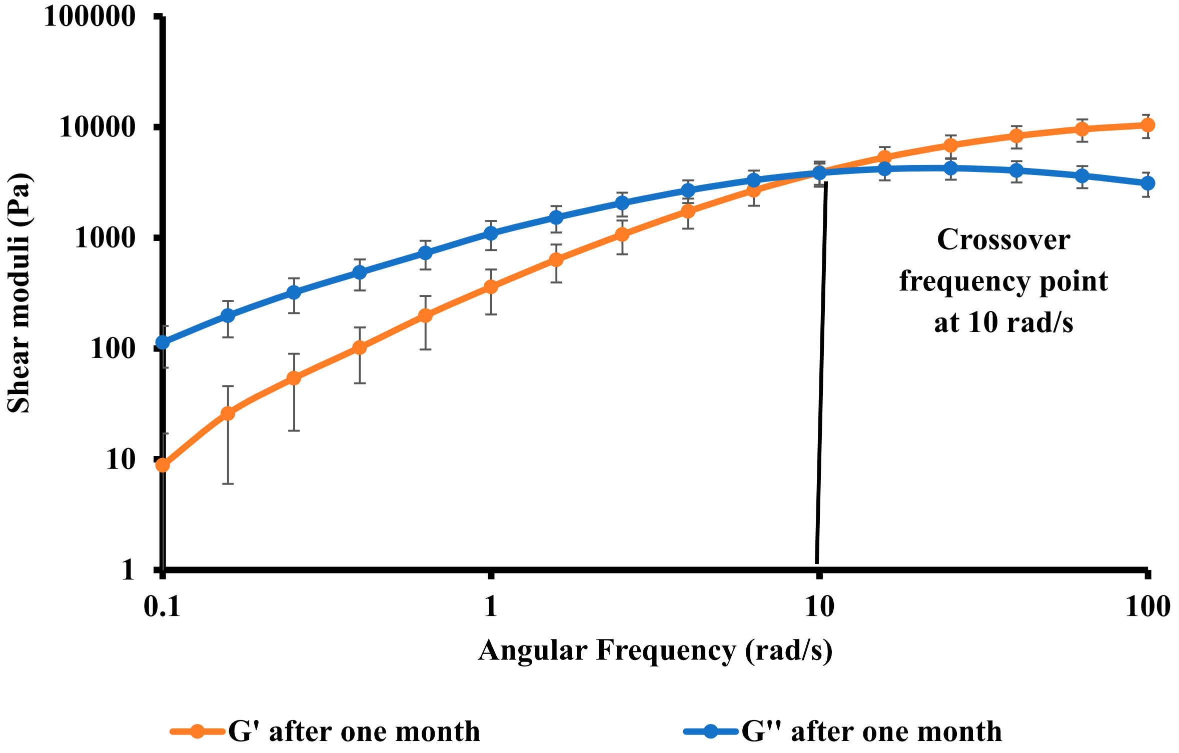

100000
10000
1000
100
10
1
Shear moduli (Pa)
0.1
1
10
100
Angular Frequency (rad/s)
Crossover frequency point at 10 rad/s
G' after one month
G'' after one month

C)

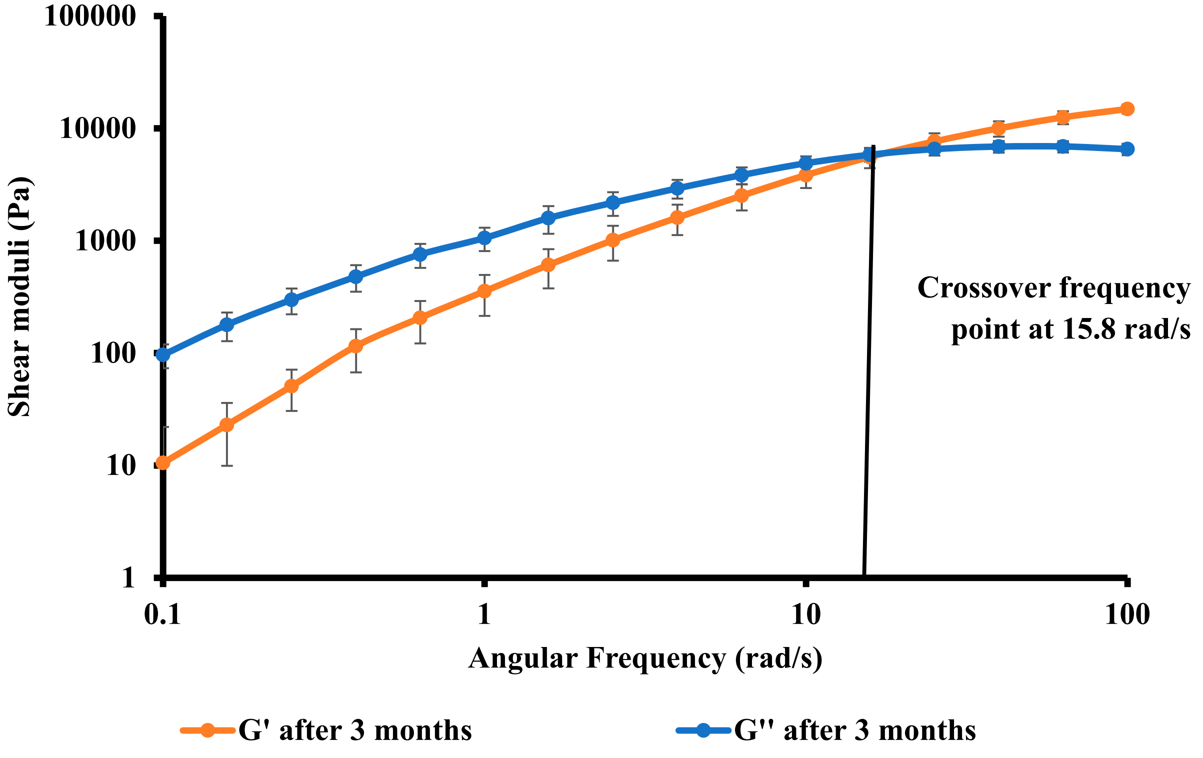

Figure S9. Crossover frequency points of CIP hydrogel patch after **(A)** one month and **(B)** three months of storage at room temperature. Data are reported as mean ± SD (n=3).
